# The importance of migratory connectivity for global ocean policy

**DOI:** 10.1098/rspb.2019.1472

**Published:** 2019-09-25

**Authors:** Daniel C. Dunn, Autumn-Lynn Harrison, Corrie Curtice, Sarah DeLand, Ben Donnelly, Ei Fujioka, Eleanor Heywood, Connie Y. Kot, Sarah Poulin, Meredith Whitten, Susanne Åkesson, Amalia Alberini, Ward Appeltans, José Manuel Arcos, Helen Bailey, Lisa T. Ballance, Barbara Block, Hannah Blondin, Andre M. Boustany, Jorge Brenner, Paulo Catry, Daniel Cejudo, Jesse Cleary, Peter Corkeron, Daniel P. Costa, Michael Coyne, Guillermo Ortuño Crespo, Tammy E. Davies, Maria P. Dias, Fanny Douvere, Francesco Ferretti, Angela Formia, David Freestone, Ari S. Friedlaender, Heidrun Frisch-Nwakanma, Christopher Barrio Froján, Kristina M. Gjerde, Lyle Glowka, Brendan J. Godley, Jacob Gonzalez-Solis, José Pedro Granadeiro, Vikki Gunn, Yuriko Hashimoto, Lucy M. Hawkes, Graeme C. Hays, Carolina Hazin, Jorge Jimenez, David E. Johnson, Paolo Luschi, Sara M. Maxwell, Catherine McClellan, Michelle Modest, Giuseppe Notarbartolo di Sciara, Alejandro Herrero Palacio, Daniel M. Palacios, Andrea Pauly, Matt Rayner, Alan F. Rees, Erick Ross Salazar, David Secor, Ana M. M. Sequeira, Mark Spalding, Fernando Spina, Sofie Van Parijs, Bryan Wallace, Nuria Varo-Cruz, Melanie Virtue, Henri Weimerskirch, Laurie Wilson, Bill Woodward, Patrick N. Halpin

**Affiliations:** 1 Nicholas School of the Environment, Duke University, Durham, NC, USA; 2 Centre for Biodiversity and Conservation Science, School of Earth and Environmental Sciences, University of Queensland, Level 5, Goddard Building (#8), St Lucia, Queensland 4072, Australia; 3 Migratory Bird Center, Smithsonian Conservation Biology Institute, National Zoological Park, Washington, DC, USA; 4 Department of Biology, Center for Animal Movement Research, Lund University, Lund, Sweden; 5 Intergovernmental Oceanographic Commission (IOC) of UNESCO, IOC Project Office for IODE, Oostende, Belgium; 6 SEO/BirdLife, Marine Programme, Barcelona, Spain; 7 Chesapeake Biological Laboratory, University of Maryland Center for Environmental Science, Solomons, MD, USA; 8 Southwest Fisheries Science Center, NOAA Fisheries, La Jolla, CA, USA; 9 Scripps Institution of Oceanography, La Jolla, CA, USA; 10 Hopkins Marine Station of Stanford University, Pacific Grove, CA, USA; 11 Monterrey Bay Aquarium, Monterey, CA, USA; 12 The Nature Conservancy, Houston, TX, USA; 13 MARE-Marine and Environmental Sciences Centre, ISPA Instituto Universitário, Lisboa, Portugal; 14 Biology Department of the University of Las Palmas de Gran Canaria, Las Palmas, Spain; 15 Protected Species Branch, NOAA Northeast Fisheries Science Center, Woods Hole, MA, USA; 16 Dept of Ecology and Evolutionary Biology, University of California Santa Cruz, Santa Cruz, CA, USA; 17 seaturtle.org, University of California Santa Cruz, Santa Cruz, CA, USA; 18 BirdLife International, Cambridge, UK; 19 UNESCO World Heritage Convention, Paris, France; 20 Department of Fish and Wildlife Conservation, College of Natural Resources and Environment, Virginia Tech, Blacksburg, VA, USA; 21 Wildlife Conservation Society, Bronx, NY, USA; Bata, Equatorial Guinea and Libreville, Gabon; 22 Sargasso Sea Commission, Washington, DC, USA; 23 Secretariat of the Convention on Migratory Species of Wild Animals, Bonn, Germany and Abu Dhabi, United Arab Emirates; 24 GOBI Secretariat, Seascape Consultants Ltd, Romsey, UK; 25 IUCN Global Marine and Polar Programme and World Commission on Protected Areas, Cambridge, MA, USA; 26 Centre for Ecology and Conservation, University of Exeter, Cornwall Campus, Penryn, UK; 27 University of Barcelona, Barcelona, Spain; 28 CESAM, Faculdade de Ciencias da Universidade de Lisboa, Lisboa, Portugal; 29 Canadian Wildlife Service, Environment and Climate Change Canada, Pacific Wildlife Research Centre, British Columbia, Canada; 30 Centre for Integrative Ecology, Deakin University, Geelong, Victoria, Australia; 31 Marviva, San José, Costa Rica; 32 University of Pisa, Pisa, Italy; 33 School of Interdisciplinary Arts and Sciences, University of Washington, Bothell Campus, Bothell, WA, USA; 34 Tethys Research Institute and IUCN Task Force on Marine Mammal Protected Areas, Milano, Italy; 35 Marine Mammal Institute and Department of Fisheries and Wildlife, Oregon State University, Newport, OR, USA; 36 Auckland War Memorial Museum, Auckland, New Zealand; 37 UWA Oceans Institute and School of Biological Sciences, Indian Ocean Marine Research Centre, University of Western Australia, Crawley, Western Australia 6009, Australia; 38 Ocean Foundation, Washington, DC, USA; 39 ISPRA—Istituto Superiore per la Protezione e la Ricerca Ambientale, Ozzano dell'Emilia, Italy; 40 Ecolibrium, Inc, Boulder, CO, USA; 41 Centre d'Etudes Biologiques de Chizé, CNRS, Villiers en Bois, France; 42 U.S. Animal Telemetry Network, NOAA/IOOS, Silver Spring, MD, USA

**Keywords:** areas beyond national jurisdiction, migratory species, marine spatial planning, area-based management

## Abstract

The distributions of migratory species in the ocean span local, national and international jurisdictions. Across these ecologically interconnected regions, migratory marine species interact with anthropogenic stressors throughout their lives. Migratory connectivity, the geographical linking of individuals and populations throughout their migratory cycles, influences how spatial and temporal dynamics of stressors affect migratory animals and scale up to influence population abundance, distribution and species persistence. Population declines of many migratory marine species have led to calls for connectivity knowledge, especially insights from animal tracking studies, to be more systematically and synthetically incorporated into decision-making. Inclusion of migratory connectivity in the design of conservation and management measures is critical to ensure they are appropriate for the level of risk associated with various degrees of connectivity. Three mechanisms exist to incorporate migratory connectivity into international marine policy which guides conservation implementation: site-selection criteria, network design criteria and policy recommendations. Here, we review the concept of migratory connectivity and its use in international policy, and describe the Migratory Connectivity in the Ocean system, a migratory connectivity evidence-base for the ocean. We propose that without such collaboration focused on migratory connectivity, efforts to effectively conserve these critical species across jurisdictions will have limited effect.

## 1. Introduction

Innovations in animal tracking technology are changing the way we think about how the world's oceans are connected [1] and about the migratory connectivity of populations and species [2]. Recent research has revealed basin-scale oceanic migrations of sea turtles, marine mammals, seabirds and fishes [3], as well as circumpolar [4] and pole-to-pole [5] migrations by seabirds. The accumulation of information about marine migratory species has been swift and massive [1]. Since 1990, over 40 000 scientific papers have been published about migratory marine species (electronic supplementary material, appendix S1 for the literature search string). Common findings from this literature highlight that many species range farther than previously known (e.g. [6]), and occur predictably at specific times in specific places, in association with specific habitats or along predictable migratory corridors [7,8].

Migratory species are increasingly exposed to the effects of a globalizing world [9], as illustrated by the spreading footprint of cumulative human impacts in the oceans [10]. The migration patterns and movements of many species span both national waters (i.e. within Exclusive Economic Zones; EEZs) and areas beyond national jurisdiction (ABNJ). A significant portion of many species' life histories are spent in the 64% of the world's oceans that lie outside national jurisdiction [11], an environment with growing human encroachment and fragmented and incomplete governance structures [12–14]. In this context, migratory species management is a complex process, often including soft law (international statements, declarations and commitments that are not legally binding but that do carry moral significance, such as United Nations resolutions including the Sustainable Development Goals), hard law (international legally binding agreements such as the United Nations Convention on the Law of the Sea (UNCLOS) and the Convention on the International Trade of Endangered Species), and multi-national consensus-based management organizations (such as the International Maritime Organization for shipping and regional fisheries management organizations for fisheries). Limitations to effective management include geographical and taxonomic gaps in governance, lack of cross-sectoral conservation tools and limited implementation of ecosystem-based approaches to management [13], as well as conservation strategies that focus on individual stages of a species migratory cycle with little consideration of population connectivity [15,16]. These limitations have hindered the development of effective management strategies for migratory species, many of which are considered at risk and in need of improved management: 95% of albatross (21 of 22 species; International Union for Conservation of Nature (IUCN) [17]), 87% of assessed migratory sharks species [18], and 63% of assessed sea turtle subpopulations (10 of 16 subpopulations; IUCN [17]) are listed as Near Threatened or Threatened (i.e. Vulnerable, Endangered or Critically Endangered) by the IUCN due primarily to indirect capture in marine fisheries (bycatch), direct harvest, predation by invasive species, or loss or degradation of habitat. Similarly, straddling (those shared between two or more jurisdictions) and highly migratory fish stocks experience twice the rate of overfishing as those within a single national jurisdiction [19].

In response to population declines of migratory species, there has been a general call for knowledge generated from animal movement data to be more effectively incorporated into management and policy frameworks (e.g. [20]). However, while the quantity of data on marine migratory species has increased dramatically in recent decades [21], efforts to synthesize and integrate information on animal movement and connectivity into global management and policy fora are nascent with examples largely originating from individual efforts [22–24]. Current mechanisms to enable discovery and access to data (e.g. data or metadata repositories) are insufficient by themselves to support global intergovernmental efforts to conserve and manage migratory species because capacity and resources for policy-makers and managers to synthesize raw data are extremely limited [25]. In this paper, we review concepts of migratory connectivity as they relate to the conservation and sustainable use of biodiversity in the oceans, with a focus on ABNJ. We provide the current status of efforts to include connectivity in management and governance approaches, and we review the information currently available to policy-makers and managers. Finally, we describe an approach that is supporting governance and management of transboundary species by aggregating, synthesizing and disseminating new knowledge on migratory connectivity in the ocean.

## 2. Definitions of migratory species differ in science and policy

Scientifically, migratory movement is commonly defined as collective, cyclical movement between separated or well-defined locations or habitats, and tends to be expressed at population or species levels [26]. These levels respectively represent the collective outcomes of individual behaviours [7], and the persistent, directed movements of an individual [27]. Existing scientific definitions include: (i) mass directional movements of large numbers of a species from one location to another [28], (ii) broad-scale movements of populations [29], (iii) movements of individuals or populations from one well-defined habitat to another, usually on temporally predictable and periodic basis [30,31], and (iv) collective movement of individuals that occurs chiefly through motivated behaviours, resulting in changed ecological status [7].

While scientific definitions usually include some measure of separation, they are commonly not restricted by the need to cross geopolitical boundaries as is often the case with established policy definitions. For example, the Convention on Migratory Species (CMS, Article I, 1.a) defines migratory species as ‘the entire population or any geographically separate part of the population of any species or lower taxon of wild animals, a significant proportion of whose members cyclically and predictably cross one or more national jurisdictional boundaries'. This definition shares two commonalities with scientific definitions by referring to collective and cyclical movements, however, it specifically requires the crossing of national boundaries. There are strong policy rationales for this requirement in the Convention's text. Primary principles of the CMS are that States are the protectors of the migratory species that live within or pass through their jurisdictions, and international cooperation of States is essential for the conservation of migratory species.

While the CMS definition itself is not explicitly limiting to taxonomic grouping, in practice, all species listed on the CMS are vertebrates with the exception of a single terrestrial invertebrate, the monarch butterfly (*Danaus plexippus*). The CMS Conference of the Parties propose and approve appendix listings, and many species, clearly migratory by scientific definitions, are not included (for example, northern elephant seals, *Mirounga angustirostris*, southern elephant seals, *Mirounga leonina* and many shearwater species, electronic supplementary material, appendix S2). To underpin an evidence-base on migratory connectivity, we developed a list of migratory megavertebrate species from multiple sources including Lascelles *et al*. [32] (*n* = 829), Fowler [18] (*n* = 94), fish species managed by Regional Fisheries Management Organizations (*n* = 40), seabirds of the U.S. Migratory Bird Treaty Act (*n* = 171) and BirdLife International (*n* = 280). After removing duplicates, the initial set of species to be included in our synthesis includes 439 fish, 346 seabird, 99 marine mammal and seven sea turtle species. The list was reviewed by taxa experts and updates to some scientific names were made based on these recommendations.

In this paper, we focus on synthesizing knowledge about migratory fishes, marine mammals, seabirds, sharks and sea turtles (electronic supplementary material, appendix S2), because these are the taxonomic groups included in the broadest policy definition represented by the CMS definition. However, we acknowledge that other marine taxa are migratory species by biological definitions and their omission from policy definitions may be detrimental to their management. Bridging the gap between science and policy definitions of migratory species is a first step in translating migratory connectivity knowledge into policy and we hope to support increasing knowledge about such species through the synthesis approach outlined below.

## 3. Understanding connectivity is crucial for the conservation and sustainable use of migratory species

Migratory species depend upon critical habitats for breeding and foraging, as well as pathways connecting these habitats. Over the course of their lives, many migratory marine species exhibit at least one of the three forms of connectivity described by Webster *et al*. [2]: (i) migratory connectivity, the seasonal movements of individuals between breeding and post-breeding foraging sites; (ii) landscape/seascape connectivity, the regional movement of individuals among habitat patches; and (iii) natal dispersal, the spread of individuals from birth sites to breeding sites.^1^ During their migrations, individuals and populations encounter a variety of stressors, from predation and adverse weather to human impacts including habitat destruction, direct and incidental fishing mortality, ship strikes, noise, hazardous substances and other pollutants [10,34]. Migratory connectivity, the geographical linking of individuals and populations throughout their migratory cycles [35] (see case study in box 1), is a major factor affecting how stressors impact individuals at each crucial life-history stage, and how these effects may scale up to effects on population abundance and distribution, and species persistence (for a worked example, see [40]). Understanding how a population is connected, how connectivity influences demographic rates, and designing conservation and management measures appropriate for the level of risk associated with various degrees of connectivity, are all critical to the conservation and sustainable use of migratory species (box 1, [41]).

Box 1. **Use of migratory connectivity knowledge in global ocean policy, a case study from the International Whaling Commission (IWC).**

To evaluate effects of incidental or directed take of cetaceans with reference to a conservation and management goal, the Scientific Committee of the IWC uses a process called the Revised Management Procedure (RMP) to estimate sustainable catch limits for commercial whaling of baleen whales. The RMP is a management strategy evaluation (simulation modelling) approach [36] that accounts for abundance, catches and population structure—including how many stocks exist and how they mix across space and time—to project population estimates under management scenarios [37]. While the RMP does not explicitly include a ‘connectivity’ parameter, connectivity information is used to inform the discussion on abundance estimates and stock structure. The evaluation process considers available evidence on movements of individuals/populations through time/area strata, for example, between breeding and feeding areas and these estimates are carried forward through simulation trials [37]. Connectivity information is therefore critical for defining model parameters, including stock structure, and for assessing differential impacts to populations of evaluated management scenarios. Regular Implementation Reviews are required to assess available evidence for abundance estimates and stock structure [37]. Migratory movements are incorporated through many data types, including photo identity, passive acoustics, genetics and satellite telemetry.

A recent example of how this works in practice is the first Implementation Review for western North Pacific Bryde's whales (*Balaenoptera brydei/edeni*), initiated in 2017 [38]. Given many data gaps for spatial and genetic structure of cetacean populations, migratory connectivity between breeding and foraging areas is often uncertain or unknown and thus multiple hypotheses of spatial stock structure are typically advanced for formal evaluation. Among multiple hypotheses of stock structure for western North Pacific Bryde's whales, two hypotheses (named hypotheses 2 and 5 by the committee) were selected as plausible given available evidence and advanced to be used in implementation simulation trials [38]. The two hypotheses clearly illustrate separate possibilities regarding migratory connectivity between breeding and feeding areas. Hypothesis 2 supports strong connectivity between breeding stocks and feeding sub-areas, i.e. individuals found in each feeding sub-area are hypothesized to be members of separate breeding stocks. By contrast, hypothesis 5 allows for mixing of individuals from the two breeding stocks in the eastern portion of sub-area 1. Both hypotheses are then considered in simulation modelling variants. In 2018, final specifications for parameters in simulation models were agreed upon by the Scientific Committee, including abundance estimates for sub-areas, mixing matrices, and future sighting survey plans and whaling options [39]. Finally, to complete the Implementation Review, the Scientific Committee (most typically through inter-sessional workshops, [37]) conducts population projections under alternative RMP variants and survey plans, results of which are intended for 2019 [39]. The processes described above are reliant on data contributions and attendance at meetings by individual experts. While this framework has generated important results, a systematic approach to aggregating, storing and disseminating knowledge of the migratory connectivity of cetacean populations (like the one described below) could greatly aid the work of the Scientific Committee.

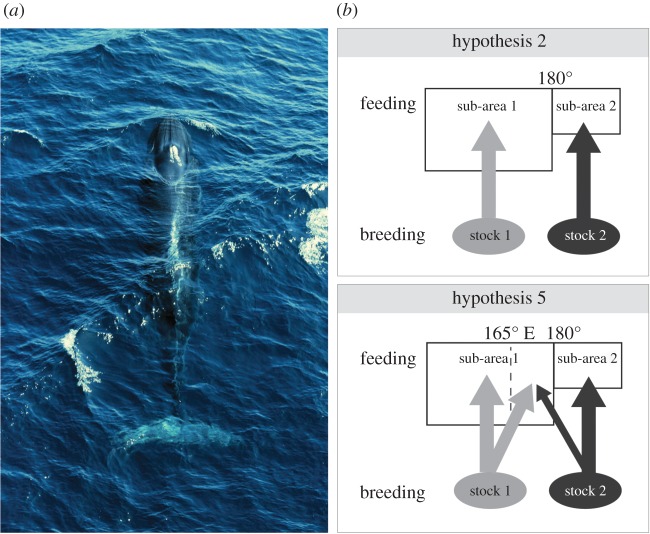

(*a*) A Bryde's whale (*Balaenoptera brydei/edeni*) surfaces in waters of the Pacific Ocean. Photo credit: David Day. (*b*) Among multiple hypotheses of western North Pacific Bryde's whale stock structure/migratory connectivity, two (named hypotheses 2 and 5) were selected as plausible given available evidence and advanced for evaluation in simulation modelling by the IWC's Scientific Committee (adapted from [38]). Hypothesis 2 demonstrates strong connectivity between breeding and feeding areas while hypothesis 5 allows for mixing of the two breeding stocks in the eastern portion of feeding sub-area 1.

The need to maintain migratory connectivity is also critical for sustaining human livelihoods and cultural connections. Migratory species provide a diverse array of cultural, regulating and provisioning ecosystem services, including contributions to aesthetic and recreational experiences, spiritual or religious enrichment, reduction of pest infestations and disease transmission, and provision of food [42]. A further regulatory service comes from the disproportionately strong influence that highly migratory species, many of which are apex predators, play in structuring of ecological communities [43]. As fishing, shipping and pollution increased in ABNJ after the 1950s [12,44], the potential for negative effects on migratory species with socioeconomic or cultural significance has increased. Groups of culturally significant species, like salmon to the indigenous coastal communities of the Pacific northwest coast of North America, and sharks to Micronesian cultures in the western South Pacific, have been impacted by activities in ABNJ—far distant from the human communities that culturally value them [45].

## 4. Connectivity has been included in area-based management approaches and governance of areas beyond national jurisdiction

Area-based management of migratory species in ABNJ is at a critical stage. The Convention on Biological Diversity (CBD) is concluding the first iteration of regional workshops to describe Ecologically or Biologically Significant Marine Areas (EBSA) [25,46,47] many of which are based on the distribution of migratory species [48]. The CBD's EBSA process encouraged the United Nations Educational, Scientific and Cultural Organization (UNESCO) to consider expanding its purview to identify World Heritage Sites in ABNJ [49]. Global fisheries and deep-sea mining authorities are continuing to develop and modify spatial management measures [50,51]. Three Regional Seas Organizations have implemented marine protected areas in ABNJ pursuant to the Convention for the Protection of the Marine Environment and the Coastal Region of the Mediterranean 1995 (Barcelona Convention); the Convention on the Conservation of Antarctic Marine Living Resources 1980 (CCAMLR) and the Convention for the Protection of the Marine Environment of the Northeast Atlantic 1992 (OSPAR Convention). At least three other Regional Seas Organizations are seeking to expand their mandate to cover ABNJ. The International Whaling Commission (IWC) uses information on migratory connectivity to improve their understanding of cetacean stock structure for their revised management process (box 1), and Regional Fisheries Management Organizations require similar information to manage fish stocks and mitigate bycatch. Finally, ongoing negotiations at the UN General Assembly (UNGA) over a treaty for the conservation and sustainable use of biodiversity in ABNJ include discussions of a global mechanism to develop cross-sectoral marine protected areas and engage in strategic environmental assessments (UNGA Resolution 72/249).

As the above policies and management regimes unfold, understanding of how migratory species fit into these frameworks is critical. Examples of the inclusion of migratory connectivity in policy can be divided into three categories: site-selection criteria, network design criteria and policy recommendations.

### (a) Site-selection criteria

Within area-based planning frameworks, focus is frequently placed on areas that are important to specific life-history stages of migratory species. For example, two of the CBD's EBSA criteria (CBD Decision IX/20 Annex I; ‘Special importance for life-history stages of species' and ‘Importance for threatened, endangered or declining species and/or habitats’) are used to describe important sites for migratory species [48]. A common justification for describing a site as meeting either criterion is the existence of an Important Bird and Biodiversity Area (IBA). IBAs are identified by BirdLife International based on the per cent of a population using a specific site. For marine birds, IBAs usually encompass important foraging habitats and are typically located around breeding grounds, or in post-breeding areas [52]. However, in specific cases, enough of a population may be geographically constrained during migration to result in a portion of a migratory corridor also being identified as an IBA [53]. The Key Biodiversity Areas (KBAs) framework, led and coordinated by a partnership of 12 conservation organizations uses a similar population threshold approach and thus may also identify portions of a migratory corridor for species in addition to seabirds, at least for those species that can easily be counted [54]. The IUCN Marine Mammal Protected Area Task Force has also developed general criteria based on expert opinion to describe Important Marine Mammal Areas (IMMAs) including Migratory Routes (sub-criterion C (iii); [55]), that are acknowledged at an intergovernmental level (UNEP/CMS Resolution 12.13)).

### (b) Network design criteria

Connectivity as a concept is often included in criteria for the development of networks of marine protected areas, most frequently in reference to larval connectivity (natal dispersal), but also in reference to migratory and seascape connectivity. For example, connectivity is not explicitly one of the site-selection criteria for EBSAs, but is one of five criteria in the CBD's scientific guidance for selecting areas to establish a representative network of marine protected areas (CBD Decision IX/20 Annex II). As a network criterion, connectivity has been used operationally to assess the ecological coherence of networks of protected areas (e.g. [56,57]).

### (c) Policy recommendations

Beyond site-selection and network criteria, intergovernmental organizations have also addressed the question of connectivity through decisions, resolutions and targets. For example, Aichi Biodiversity Target 11 calls for 10% of coastal and marine areas to be conserved through, *inter alia*, ‘well-connected systems of protected areas'. The CMS has gone further in describing connectivity and migratory connectivity by adopting resolutions on ecological networks (consolidated and updated in 2017; UNEP/CMS/Resolution 12.7) and the importance of including migratory connectivity in conservation decisions (UNEP/CMS/Resolution 12.26). Resolution 12.7 recommends making connectivity between important areas explicit.

4. Encourages Parties and other Range States, when identifying areas of importance to migratory terrestrial, avian and aquatic species, to take into account and make explicit by description, schematic maps or conceptual models the relationship between those areas and other areas which may be ecologically linked to them, in physical terms, for example as connecting corridors, or in other ecological terms, for example as breeding areas related to non-breeding areas, stopover sites, feeding and resting places (CMS 2017).

While the concept of connectivity is ubiquitous in multilateral environmental agreements, the ‘schematic maps or conceptual models' recommended by CMS are still rare. A lack of easily accessible and usable [58] geospatial information prevents the full consideration of migratory connectivity in area-based planning processes [25], and limits the ability to conduct meaningful environmental impact assessments and strategic environmental assessments. Further, these types of maps and models are necessary to achieve United Nations Sustainable Development Goal 14 as they inform sustainable management of coastal and marine ecosystems (14.2), underpin fisheries models required to end overfishing (14.4), support development of area-based management tools (14.5), provide economic benefit to Small Island Developing States that depend on migratory species (14.7), and increase scientific knowledge, capacity development and technology transfer (14.A). Mainstreaming marine biodiversity into the United Nations Sustainable Development Goals will require integration of migratory connectivity information and its application to ‘other effective conservation measures' such as sectoral ‘*in-situ*’ efforts to conserve biodiversity [59].

Access to baseline information on migratory connectivity in the ocean will become even more important for the development of area-based management tools and conservation planning under future climate change scenarios. Species that migrate between breeding and feeding habitats can be strongly affected by climate change. For example, climate change could easily disrupt cross-environment correlations that make migration routes and timing adaptive to environmental cues [60], potentially altering connectivity patterns and in turn, the effectiveness of protected areas. Johnson *et al*. [61] considered the interaction of climate change on EBSAs and existing area-based management tools in the North Atlantic Ocean, concluding that altered patterns of connectivity related to climate change can be expected to influence the effectiveness of marine protected areas. Developing baselines now and monitoring changes in migratory connectivity through time will be critical to plan for future changes.

The parties to the CMS recognized this need (i.e. to aggregate and synthesize information on migratory connectivity) and in 2017 encouraged ‘support for the enhancement of the databases… [and] targeted joint analyses of animal movements and other factors using these databases in an integrated way across the marine and terrestrial realms so as to improve understanding of the biological basis of migratory species connectivity’ (UNEP/CMS/Resolution 12.26). Below, we consider in more depth what databases and information are currently available.

## 5. What information on marine migratory connectivity is available to policy-makers and managers?

### (a) Species distribution

Products with spatial information on species distribution are currently available (electronic supplementary material, Appendix S3). The Global Register of Migratory Species (GROMS) summarized the state of knowledge on the distribution of migratory species globally [62,63]. GROMS is a relational database containing distribution data for 2880 (terrestrial and marine) vertebrate migratory animals, last updated in 2004. IUCN and BirdLife International both provide range maps of species. AquaMaps provides modelled range and distribution maps of many migratory marine species. The State of the World's Sea Turtles (SWOT) has compiled distribution information on the world's seven sea turtle species from literature and expert opinion. However, the spatial resolution of distribution and range maps in GROMS, BirdLife's Datazone, the IUCN Redlist, AquaMaps and the SWOT database lack the spatial, temporal and ecological resolution necessary to inform the development of specific area-based management measures, and they do not contain information on migration pathways or migratory connectivity.

### (b) Electronic tracking data

The most readily accessible information for directly informing area-based management (e.g. animal location data provided by acoustic and satellite telemetry and other types of electronic animal tracking devices) rest in data repositories, e.g. the United States' Animal Telemetry Network, Australia's Integrated Marine Observing System Animal Tracking Database, BirdLife International's Seabird Tracking Database, OBIS-SEAMAP, the Ocean Tracking Network, Movebank or the satellite tracking and analysis tool of seaturtle.org (see the electronic supplementary material, Appendix S3). However, for most decision-making purposes, a substantial amount of work is required to make practical use of tracking data. Considerations including deployment location, sample size, location error and representativeness of life-history stages, sexes, populations and species need to be assessed and treated appropriately prior to use which requires analytical expertise and time [20]. The need to process and analyse tracking data prior to including them within decision-making frameworks has limited their use to date in existing area-based planning and management processes.

### (c) Derived products focusing on important sites

Taxa-specific and/or regional efforts to summarize information pertaining to migratory connectivity have been published (e.g. [64]), but have rarely been developed into open-access knowledge systems for use by marine spatial planning processes. BirdLife International provides synthesized telemetry data products to management and policy arenas via its identification of marine IBAs. The existence of a freely accessible database of IBAs has resulted in stronger uptake and application of this information in the CBD's regional EBSA workshops compared to other taxonomic groups that do not have such knowledge summarized and available [25]. Analogous efforts for marine mammals (IMMAs, [65]), sea turtles (SWOT's Global Sea Turtle Tracking Initiative), sharks (e.g. the Global Shark Movement Project; [66]) and across taxa (e.g. the Marine Megafauna Movement Analytical Program (MMMAP); [21]) share a similar vision.

## 6. The Migratory Connectivity in the Ocean system combines efforts to move from data to usable knowledge

**Figure 1.**
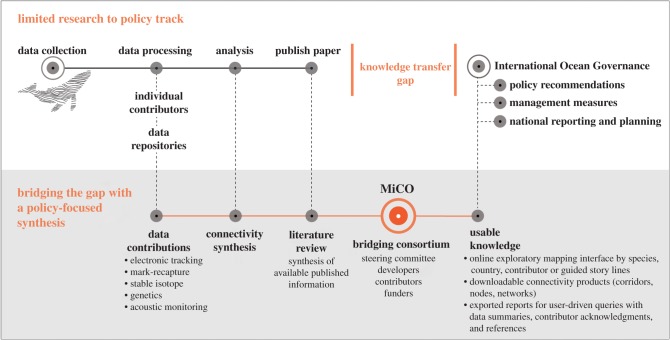
MiCO bridges a knowledge communications gap between researchers and policy fora. The typical flow of knowledge from data collection to scientific publication limits access to that knowledge and is dependent on participation by each individual researcher in all relevant policy processes. Bridging consortia like MiCO provide mechanisms to increase access to knowledge, ensure that it is provided in a usable format, and allow contributors to track the impact of their work.

The efforts described above provide critical services as data and information brokers, but none provide usable geospatial knowledge on migratory connectivity to management and policy arenas. The many intergovernmental management and policy processes we reviewed above seek to use information about migratory species, suggesting that an evidence-base of accessible, easily interpreted and synthesized knowledge on the topic could have a large policy impact. However, there are obstacles to effective knowledge transfer between scientists and policy-makers, including ‘differences between researchers and policy-makers in their cultures, time-frames, reward structures, and motivations’, and the need for better mechanisms ‘to ensure uptake of research that is intended to be policy-relevant’ [67]. In response to this need, a consortium of data repositories, national observing systems, taxa conservation groups, museums, environmental non-governmental organizations (NGOs), universities, individuals, intergovernmental organizations and UN bodies, many of which initiated processes and created tools described in this paper above, have now developed a global open-access online system providing usable knowledge about migratory connectivity in the ocean (MiCO: www.mico.eco). The MiCO consortium designed the system to be a bridge between individuals/organizations generating data or products that describe migratory connectivity and policy fora or management organizations engaged in marine resource management, conservation, spatial planning and environmental assessment processes (figure 1).

To impact and support global ocean policy efforts, the evidence-base that MiCO is creating: (i) provides additional value to both data/product contributors, and policy-makers and managers; (ii) complements the strengths of existing data repositories and research programmes globally; (iii) integrates information across data types, primary scientific literature, traditional knowledge and expert opinion; (iv) facilitates the delivery of those products to management organizations and policy processes in a transparent manner with explicit acknowledgement of contributors; and, (v) is easily accessible, freely available and updated on time-scales relevant to policy and management requirements. To ensure that the system provides value to both contributors and users, advisory panels were developed including: (i) strategic (longer-term goal setting) and technical (frequent input on methods and implementation) advisory panels populated with leading researchers from academia, government agencies and data repositories and (ii) a policy advisory panel including staff from CMS (and its family of instruments), CBD, UNESCO, OBIS, IUCN, the Sargasso Sea Commission and NGOs.

MiCO integrates information across data types by aggregating and synthesizing available information about migratory connectivity through a comprehensive literature review, aggregating existing data and synthetic products from contributors, and creating new synthetic products to serve policy processes. Six types of data can be used to derive products to help describe migratory connectivity: electronic tracking data, capture–mark–recapture, observations including visual surveys, data on stable isotope ratios, population genetics and passive acoustic monitoring. Each of the methodologies has unique characteristics, provides information at different spatial and temporal scales, and may be better suited for species with different traits. To serve as an evidence-base for available scientific information on the migratory connectivity of marine species, the systematic literature review reduces biases by: (i) the transparent development of a list of migratory megavertebrate species to be included (described above and in the electronic supplementary material, Appendix S2); (ii) vetting this list with species experts (many of the co-authors of this paper); (iii) creating and testing taxa-specific search strings for sensitivity in results returned; (iv) conducting the searches over a short period to avoid biases in literature availability; and (v) ensuring an exhaustive search by conducting searches on two separate large, multi-disciplinary indexing and citation databases (Web of Science and SCOPUS), as well as a separate manual searches on an un-indexed and highly relevant journal (Animal Biotelemetry). While our search was systematic and exhaustive for English-language literature, broadening the evidence-base to include non-English sources is a future goal. The initial focus of MiCO analyses has been the development of products from this literature review and from electronic tracking data, provided either directly by data holders or through literature review, with the intent to add additional data types in the future to underpin network diagrams illustrating directionality and strength of connectivity among use areas.

To facilitate delivery of migratory connectivity knowledge to policy and management arenas, a prototype system (www.mico.eco/system) was developed to provide a basis for contributors and policy-makers to offer feedback on methods and usability. The system was launched in April 2019 at the second UN Intergovernmental Conference on a new international treaty for the conservation and sustainable use of marine biological diversity of ABNJ. The prototype includes a set of 38 standardized area-use models describing general and core-use areas for a given population/species by activity (e.g. breeding, migrating, non-breeding, ranging, etc.; see www.mico.eco/methods for detail) for 357 animals from seven species across 55 EEZs. To provide this knowledge in as transparent a manner as possible, metadata describing datasets, animals, activity and (if known) population, sex, age class and data density are graphically presented by month and year for each area or population/species. System users can view summaries of area use by species, by a country's EEZ or relative to ABNJ, and review summaries of data contributed per provider, with associated references created from the data. An interactive mapper allows detailed geographical exploration, examination and overlays of animal use areas filtered by any combination of available metadata. Species range maps, when available, are layered under use areas to describe the degree of coverage of MiCO products.

These MiCO products, metadata from the literature review and cases studies have already informed the work of three regional seas organizations (Nairobi Convention, Abidjan Convention and the Comisión Permanente del Pacific Sur), the CMS in their development of resolution 12.26, and considerations of area-based management tools, environmental impact assessments and capacity building and technology transfer in the negotiations for a new treaty for marine biodiversity in ABNJ. A key component of the system is its ability to track how contributor data have been used in these arenas, providing individual researchers the ability to assess and report their impact on management and policy fora. This provides incentive for researchers to contribute data to policy processes and measure their impact by doing so, while at the same time, the system protects raw data that may not yet be available for public sharing by disseminating only synthesized products.

Support for the system's development and for outreach to intergovernmental organizations has come from a national government (the International Climate Initiative of the German Federal Ministry for the Environment, Nature Conservation and Nuclear Safety via the Global Ocean Biodiversity Initiative [68]), and from two components of the Global Environment Facility's Sustainable Fisheries Management and Biodiversity Conservation of Deep-sea Living Marine Resources and Ecosystems in the ABNJ project. Future support for the system is likely to come in two phases. The first phase, dedicated to development of a data-rich system with greater functionality, will probably come from similar sources via grants to academic institutions. The second phase, focused on maintenance and better tailoring to support national reporting and global indicators, would see the system transferred to an intergovernmental organization with a budget for structural support of the system. The history of OBIS presents a direct analogue for this life cycle, beginning as a programme of the Census of Marine Life and then was transferred to within UNESCO-IOC [69].

MiCO seeks to build on its strong foundation and invites engagement from additional stakeholders to inform its continued development and enhancement to best serve both users and contributors. Strong forward momentum in global ocean governance will soon result in major policy changes for the ocean with many management implications for migratory marine species. By integrating and scaling up the information gained through the tens of thousands of scientific papers about migratory species into knowledge that is relevant and usable in this new era of ocean policy, we hope to make a step-change in evidence-based policy to conserve migratory marine species.

## Supplementary Material

Appendices 1-3

## Endnote

1 In marine ecology, larval connectivity/dispersal from spawning to settlement site is a form of natal dispersal, while the term marine population connectivity refers to the exchange of individuals among geographically separated breeding subpopulations (i.e. in a permanent rather than periodic/migratory sense to subsequently influence population genetics, rates of immigration into populations and spread of invasive species) [33].

## Data accessibility

All products from the Migratory Connectivity in the Ocean (MiCO) system are freely available online at mico.eco/system.
