# Supplementary material for: The importance of migratory connectivity for global ocean policy
Source: Proc Biol Sci. 2019 Sep 25;286(1911):20191472. doi: 10.1098/rspb.2019.1472 (PMC6784718; doi:10.1098/rspb.2019.1472)
Supplement: Appendices 1-3 [file rspb20191472supp1.pdf]

Appendix 1: Search string to identify papers related to migratory connectivity in the ocean

This is the information returned from a search of the Web of Science database to understand the accumulation of information about marine migratory species since 1990.

Web of Science Search, conducted January 29, 2019

Results: 40,254

(from All Databases)

You searched for: TOPIC: ("marine" and "animal" and (telemetry or tag or isotop* or genetic* or mark or recapture) and (migrat* or connect* or move* or feed* or forag* or breed* or dispers* or nest* or aggregat* or ground* or site* or corridor or route* or track* or winter* or habitat*)) AND YEAR PUBLISHED: (1990-2019)*

Refined by: LANGUAGES: (ENGLISH)

Timespan: 1990-2019. Databases: WOS, BCI, CCC, DRCI, DIIDW, KJD, MEDLINE, RSCI, SCIELO, ZOOREC.

Appendix 2: Species List

MiCO aims to coalesce existing knowledge on connectivity and migratory corridors of marine migratory species that utilize areas beyond national jurisdiction. Numerous definitions of "migration" and "migratory species" exist, confusing efforts to develop a specific list of species to include within MiCO. Lascelles et al. (2014) assessed the current status and management needs of over 800 migratory marine species across the same four taxonomic groups as MiCO. They define migratory species as animals that move between "at least two jurisdictions during the course of their annual cycles," with specific species selected by expert groups. The list of species to be evaluated by MiCO was assembled from several sources: including Lascelles et al. (2014) (n = 829), the CMS Migratory Shark Species (n = 94), fish species managed by Regional Fisheries Management Organization (RFMO; n = 40), seabirds of the Migratory Bird Treaty Act (n = 171), and BirdLife International (n = 280). The initial 892 species to be evaluated by the MiCO system includes 440 fish, 346 seabird, 99 marine mammal, and 7 sea turtle species.

	Family	Scientific Name	Common Name	CMS Annex (as of Aug 7 2018)	IUCN Red List Status
1	Balaenidae	Balaena mysticetus	Bowhead Whale	I	LC
2	Balaenidae	Eubalaena australis	Southern Right Whale	I	LC
3	Balaenidae	Eubalaena glacialis	North Atlantic Right Whale	I	EN
4	Balaenidae	Eubalaena japonica	North Pacific Right Whale	I	EN
5	Balaenopteridae	Balaenoptera acutorostrata	(Common) Minke Whale		LC
6	Balaenopteridae	Balaenoptera bonaerensis	Antarctic Minke Whale	II	NT
7	Balaenopteridae	Balaenoptera borealis	Sei Whale	I/II	EN
8	Balaenopteridae	Balaenoptera edeni	Bryde's Whale	II	LC
9	Balaenopteridae	Balaenoptera musculus	Blue Whale	I	EN
10	Balaenopteridae	Balaenoptera omurai	Omura,Äôs Whale	II	DD
11	Balaenopteridae	Balaenoptera physalus	Fin Whale	I/II	EN
12	Balaenopteridae	Megaptera novaeangliae	Humpback Whale	I	LC
13	Delphinidae	Cephalorhynchus heavisidii	Heaviside's Dolphin	II	DD
14	Delphinidae	Delphinus capensis	Long-beaked Common Dolphin		DD
15	Delphinidae	Delphinus delphis	Short-beaked Common Dolphin	I/II	LC
16	Delphinidae	Feresa attenuata	Pygmy Killer Whale		DD
17	Delphinidae	Globicephala macrorhynchus	Short-finned Pilot Whale		DD
18	Delphinidae	Globicephala melas	Long-finned Pilot Whale	II	DD
19	Delphinidae	Grampus griseus	Risso's Dolphin	II	LC
20	Delphinidae	Lagenodelphis hosei	Fraser's Dolphin	II	LC
21	Delphinidae	Lagenorhynchus acutus	Atlantic White-sided Dolphin	II	LC
22	Delphinidae	Lagenorhynchus albirostris	White-beaked Dolphin	II	LC
23	Delphinidae	Lagenorhynchus australis	Peale's Dolphin	II	DD
24	Delphinidae	Lagenorhynchus cruciger	Hourglass Dolphin		LC
25	Delphinidae	Lagenorhynchus obliquidens	Pacific White-sided Dolphin		LC
26	Delphinidae	Lagenorhynchus obscurus	Dusky Dolphin	II	DD
27	Delphinidae	Lissodelphis borealis	Northern Right Whale Dolphin		LC
28	Delphinidae	Lissodelphis peronii	Southern Right Whale Dolphin		DD
29	Delphinidae	Orcinus orca	Killer Whale	II	DD
30	Delphinidae	Peponocephala electra	Melon-headed Whale		LC
31	Delphinidae	Pseudorca crassidens	False Killer Whale		DD
32	Delphinidae	Stenella attenuata	Pantropical Spotted Dolphin	II	LC
33	Delphinidae	Stenella clymene	Clymene Dolphin	II	DD
34	Delphinidae	Stenella coeruleoalba	Striped Dolphin	II	LC
35	Delphinidae	Stenella frontalis	Atlantic Spotted Dolphin		DD
36	Delphinidae	Stenella longirostris	Spinner Dolphin	II	DD
37	Delphinidae	Steno bredanensis	Rough-toothed Dolphin		LC
38	Delphinidae	Tursiops aduncus	Indo-Pacific Bottlenose Dolphin	II	DD
39	Delphinidae	Tursiops truncatus	Common Bottlenose Dolphin	I/II	LC
40	Dugongidae	Dugong dugon	Dugong	II	VU
41	Eschrichtiidae	Eschrichtius robustus	Gray Whale		LC

	Family	Scientific Name	Common Name	CMS Annex (as of Aug 7 2018)	IUCN Red List Status
42	Monodontidae	Delphinapterus leucas	Beluga Whale	II	LC
43	Monodontidae	Monodon monoceros	Narwhal	II	LC
44	Neobalaenidae	Caperea marginata	Pygmy Right Whale	II	DD
45	Odobenidae	Odobenus rosmarus	Walrus		VU
46	Otariidae	Arctocephalus australis	South American Fur Seal	II	LC
47	Otariidae	Arctocephalus forsteri	New Zealand Fur Seal		LC
48	Otariidae	Arctocephalus gazella	Antarctic Fur Seal		LC
49	Otariidae	Arctocephalus philippii	Juan Fern√°ndez Fur Seal		LC
50	Otariidae	Arctocephalus pusillus	Afro-Australian Fur Seal		LC
51	Otariidae	Arctocephalus townsendi	Guadalupe Fur Seal		LC
52	Otariidae	Arctocephalus tropicalis	Subantarctic Fur Seal		LC
53	Otariidae	Callorhinus ursinus	Northern Fur Seal		VU
54	Otariidae	Zalophus californianus	Californian Sea Lion		LC
55	Phocidae	Cystophora cristata	Hooded Seal		VU
56	Phocidae	Erignathus barbatus	Bearded Seal		LC
57	Phocidae	Halichoerus grypus	Grey Seal	II	LC
58	Phocidae	Hydrurga leptonyx	Leopard Seal		LC
59	Phocidae	Leptonychotes weddellii	Weddell Seal		LC
60	Phocidae	Lobodon carcinophaga	Crabeater Seal		LC
61	Phocidae	Mirounga angustirostris	Northern Elephant Seal		LC
62	Phocidae	Mirounga leonina	Southern Elephant Seal		LC
63	Phocidae	Ommatophoca rossii	Ross Seal		LC
64	Phocidae	Pagophilus groenlandicus	Harp Seal		LC
65	Phocidae	Phoca largha	Spotted Seal		LC
66	Phocidae	Pusa hispida	Ringed Seal		LC
67	Phocoenidae	Neophocaena asiaeorientalis	Narrow-ridged Finless Porpoise	II	EN
68	Phocoenidae	Neophocaena phocaenoides	Indo-Pacific Finless Porpoise	II	VU
69	Phocoenidae	Phocoena dioptrica	Spectacled Porpoise	II	DD
70	Phocoenidae	Phocoena spinipinnis	Burmeister's Porpoise	II	DD
71	Phocoenidae	Phocoenoides dalli	Dall's Porpoise	II	LC
72	Physeteridae	Kogia breviceps	Pygmy Sperm Whale		DD
73	Physeteridae	Kogia sima	Dwarf Sperm Whale		DD
74	Physeteridae	Physeter macrocephalus	Sperm Whale	I/II	VU
75	Trichechidae	Trichechus inunguis	South American Manatee	II	VU
76	Trichechidae	Trichechus manatus	American Manatee	I/II	VU
77	Trichechidae	Trichechus senegalensis	African Manatee	I/II	VU
78	Ursidae	Ursus maritimus	Polar Bear	II	VU
79	Ziphiidae	Berardius arnuxii	Arnoux's Beaked Whale		DD
80	Ziphiidae	Berardius bairdii	Baird's Beaked Whale	II	DD
81	Ziphiidae	Hyperoodon ampullatus	North Atlantic Bottlenose Whale	II	DD
82	Ziphiidae	Hyperoodon planifrons	Southern Bottlenose Whale		LC

	Family	Scientific Name	Common Name	CMS Annex (as of Aug 7 2018)	IUCN Red List Status
83	Ziphiidae	Indopacetus pacificus	Indo-pacific Beaked Whale		DD
84	Ziphiidae	Mesoplodon bidens	Sowerby's Beaked Whale		DD
85	Ziphiidae	Mesoplodon bowdoini	Andrew's Beaked Whale		DD
86	Ziphiidae	Mesoplodon carlhubbsi	Hubbs' Beaked Whale		DD
87	Ziphiidae	Mesoplodon densirostris	Blainville's Beaked Whale		DD
88	Ziphiidae	Mesoplodon europaeus	Gervais' Beaked Whale		DD
89	Ziphiidae	Mesoplodon ginkgodens	Ginkgo-toothed Beaked Whale		DD
90	Ziphiidae	Mesoplodon grayi	Gray's Beaked Whale		DD
91	Ziphiidae	Mesoplodon hectori	Hector's Beaked Whale		DD
92	Ziphiidae	Mesoplodon layardii	Strap-toothed Whale		DD
93	Ziphiidae	Mesoplodon mirus	True's Beaked Whale		DD
94	Ziphiidae	Mesoplodon perrini	Perrin's Beaked Whale		DD
95	Ziphiidae	Mesoplodon peruvianus	Pygmy Beaked Whale		DD
96	Ziphiidae	Mesoplodon stejnegeri	Stejneger's Beaked Whale		DD
97	Ziphiidae	Mesoplodon traversii	Spade-toothed Whale		DD
98	Ziphiidae	Tasmacetus shepherdi	Shepherd's Beaked Whale		DD
99	Ziphiidae	Ziphius cavirostris	Cuvier's Beaked Whale	I	LC

	Family	Scientific Name	Common Name	CMS Annex (as of Aug 7 2018)	IUCN Red List Status
1	Alcidae	Aethia cristatella	Crested Auklet		LC
2	Alcidae	Aethia psittacula	Parakeet Auklet		LC
3	Alcidae	Aethia pusilla	Least Auklet		LC
4	Alcidae	Aethia pygmaea	Whiskered Auklet		LC
5	Alcidae	Alca torda	Razorbill		NT
6	Alcidae	Alle alle	Little Auk		LC
7	Alcidae	Brachyramphus brevirostris	Kittlitz's Murrelet		NT
8	Alcidae	Brachyramphus marmoratus	Marbled Murrelet		EN
9	Alcidae	Brachyramphus perdix	Long-billed Murrelet		NT
10	Alcidae	Cepphus carbo	Spectacled Guillemot		LC
11	Alcidae	Cepphus columba	Pigeon Guillemot		LC
12	Alcidae	Cepphus grylle	Black Guillemot		LC
13	Alcidae	Cerorhinca monocerata	Rhinoceros Auklet		LC
14	Alcidae	Fratercula arctica	Atlantic Puffin		VU
15	Alcidae	Fratercula cirrhata	Tufted Puffin		LC
16	Alcidae	Fratercula corniculata	Horned Puffin		LC
17	Alcidae	Ptychoramphus aleuticus	Cassin's Auklet		NT
18	Alcidae	Synthliboramphus antiquus	Ancient Murrelet		LC
19	Alcidae	Synthliboramphus craveri	Craveri's Murrelet		VU
20	Alcidae	Synthliboramphus hypoleucus	Guadalupe Murrelet		EN
21	Alcidae	Synthliboramphus wumizusume	Japanese Murrelet	I	VU
22	Alcidae	Uria aalge	Common Murre		LC
23	Alcidae	Uria lomvia	Thick-billed Murre		LC
24	Anatidae	Aythya marila	Greater Scaup	II	LC
25	Anatidae	Bucephala clangula	Common Goldeneye	II	LC
26	Anatidae	Bucephala islandica	Barrow's Goldeneye	II	LC
27	Anatidae	Clangula hyemalis	Long-tailed Duck	II	VU
28	Anatidae	Histrionicus histrionicus	Harlequin Duck	II	LC
29	Anatidae	Melanitta americana	Black Scoter		NT
30	Anatidae	Melanitta deglandi	White-winged Scoter		LC
31	Anatidae	Melanitta fusca	Velvet Scoter	II	VU
32	Anatidae	Melanitta nigra	Common Scoter	II	LC
33	Anatidae	Melanitta perspicillata	Surf Scoter	II	LC
34	Anatidae	Melanitta stejnegeri	Siberian Scoter		LC
35	Anatidae	Mergus merganser	Goosander	II	LC
36	Anatidae	Mergus serrator	Red-breasted Merganser	II	LC
37	Anatidae	Polysticta stelleri	Steller's Eider	I/II	VU
38	Anatidae	Somateria fischeri	Spectacled Eider	II	LC
39	Anatidae	Somateria mollissima	Common Eider	II	NT
40	Anatidae	Somateria spectabilis	King Eider	II	LC
41	Anatidae	Tachyeres patachonicus	Flying Steamerduck		LC

	Family	Scientific Name	Common Name	CMS Annex (as of Aug 7 2018)	IUCN Red List Status
42	Diomedeidae	Diomedea amsterdamensis	Amsterdam Albatross	I	CR
43	Diomedeidae	Diomedea antipodensis	Antipodean Albatross	II	EN
44	Diomedeidae	Diomedea dabbenena	Tristan Albatross	II	CR
45	Diomedeidae	Diomedea epomophora	Southern Royal Albatross	II	VU
46	Diomedeidae	Diomedea exulans	Wandering Albatross	II	VU
47	Diomedeidae	Diomedea sanfordi	Northern Royal Albatross	II	EN
48	Diomedeidae	Phoebastria albatrus	Short-tailed Albatross	I	VU
49	Diomedeidae	Phoebastria immutabilis	Laysan Albatross	II	NT
50	Diomedeidae	Phoebastria irrorata	Waved Albatross	II	CR
51	Diomedeidae	Phoebastria nigripes	Black-footed Albatross	II	NT
52	Diomedeidae	Phoebetria fusca	Sooty Albatross	II	EN
53	Diomedeidae	Phoebetria palpebrata	Light-mantled Albatross	II	NT
54	Diomedeidae	Thalassarche bulleri	Buller's Albatross	II	NT
55	Diomedeidae	Thalassarche carteri	Indian Yellow-nosed Albatross	II	EN
56	Diomedeidae	Thalassarche cauta	Shy Albatross	II	NT
57	Diomedeidae	Thalassarche chlororhynchos	Atlantic Yellow-nosed Albatross	II	EN
58	Diomedeidae	Thalassarche chrysostoma	Grey-headed Albatross	II	EN
59	Diomedeidae	Thalassarche eremita	Chatham Albatross	II	VU
60	Diomedeidae	Thalassarche impavida	Campbell Albatross	II	VU
61	Diomedeidae	Thalassarche melanophris	Black-browed Albatross	II	LC
62	Diomedeidae	Thalassarche salvini	Salvin's Albatross	II	VU
63	Diomedeidae	Thalassarche steadi	White-capped Albatross	II	NT
64	Fregatidae	Fregata andrewsi	Christmas Frigatebird	I	CR
65	Fregatidae	Fregata aquila	Ascension Frigatebird		VU
66	Fregatidae	Fregata ariel	Lesser Frigatebird		LC
67	Fregatidae	Fregata magnificens	Magnificent Frigatebird		LC
68	Fregatidae	Fregata minor	Great Frigatebird		LC
69	Gaviidae	Gavia adamsii	Yellow-billed Loon	II	NT
70	Gaviidae	Gavia arctica	Arctic Loon		LC
71	Gaviidae	Gavia immer	Common Loon		LC
72	Gaviidae	Gavia pacifica	Pacific Loon		LC
73	Gaviidae	Gavia stellata	Red-throated Loon	II	LC
74	Hydrobatidae	Hydrobates castro	Band-rumped Storm-petrel		LC
75	Hydrobatidae	Hydrobates furcatus	Fork-tailed Storm-petrel		LC
76	Hydrobatidae	Hydrobates homochroa	Ashy Storm-petrel		EN
77	Hydrobatidae	Hydrobates hornbyi	Ringed Storm-petrel		DD
78	Hydrobatidae	Hydrobates leucorhous	Leach's Storm-petrel		VU
79	Hydrobatidae	Hydrobates macrodactylus	Guadalupe Storm-petrel		CR
80	Hydrobatidae	Hydrobates markhami	Markham's Storm-petrel		DD
81	Hydrobatidae	Hydrobates matsudairae	Matsudaira's Storm-petrel		VU
82	Hydrobatidae	Hydrobates melania	Black Storm-petrel		LC

	Family	Scientific Name	Common Name	CMS Annex (as of Aug 7 2018)	IUCN Red List Status
83	Hydrobatidae	Hydrobates microsoma	Least Storm-petrel		LC
84	Hydrobatidae	Hydrobates monorhis	Swinhoe's Storm-petrel		NT
85	Hydrobatidae	Hydrobates monteiroi	Monteiro's Storm-petrel		VU
86	Hydrobatidae	Hydrobates pelagicus	European Storm-petrel		LC
87	Hydrobatidae	Hydrobates tethys	Wedge-rumped Storm-petrel		LC
88	Hydrobatidae	Hydrobates tristrami	Tristram's Storm-petrel		NT
89	Laridae	Anous minutus	Black Noddy		LC
90	Laridae	Anous stolidus	Brown Noddy		LC
91	Laridae	Anous tenuirostris	Lesser Noddy		LC
92	Laridae	Chlidonias albostriatus	Black-fronted Tern		EN
93	Laridae	Chlidonias hybridus			NA
94	Laridae	Chlidonias leucopterus	White-winged Tern	II	LC
95	Laridae	Chlidonias niger	Black Tern	II	LC
96	Laridae	Creagrus furcatus	Swallow-tailed Gull		LC
97	Laridae	Gelochelidon macrotarsa	Australian Gull-billed Tern		LC
98	Laridae	Gelochelidon nilotica	Common Gull-billed Tern	II	LC
99	Laridae	Gygis alba	Common White Tern		LC
100	Laridae	Gygis microrhyncha	Little White Tern		LC
101	Laridae	Hydrocoloeus minutus	Little Gull		LC
102	Laridae	Hydroprogne caspia	Caspian Tern	II	LC
103	Laridae	Larosterna inca	Inca Tern		NT
104	Laridae	Larus argentatus	European Herring Gull		LC
105	Laridae	Larus atlanticus	Olrog's Gull	I	NT
106	Laridae	Larus atricilla	Laughing Gull		LC
107	Laridae	Larus audouinii	Audouin's Gull	I/II	LC
108	Laridae	Larus belcheri	Belcher's Gull		LC
109	Laridae	Larus brunnicephalus	Brown-headed Gull		LC
110	Laridae	Larus bulleri	Black-billed Gull		EN
111	Laridae	Larus cachinnans	Caspian Gull		LC
112	Laridae	Larus californicus	California Gull		LC
113	Laridae	Larus canus	Mew Gull		LC
114	Laridae	Larus cirrocephalus	Grey-headed Gull		LC
115	Laridae	Larus crassirostris	Black-tailed Gull		LC
116	Laridae	Larus delawarensis	Ring-billed Gull		LC
117	Laridae	Larus dominicanus	Kelp Gull		LC
118	Laridae	Larus fuliginosus	Lava Gull		VU
119	Laridae	Larus fuscus	Lesser Black-backed Gull		LC
120	Laridae	Larus genei	Slender-billed Gull	II	LC
121	Laridae	Larus glaucescens	Glaucous-winged Gull		LC
122	Laridae	Larus glaucoides	Iceland Gull		LC
123	Laridae	Larus hartlaubii	Hartlaub's Gull		LC

	Family	Scientific Name	Common Name	CMS Annex (as of Aug 7 2018)	IUCN Red List Status
124	Laridae	Larus heermanni	Heermann's Gull		NT
125	Laridae	Larus hemprichii	Sooty Gull	II	LC
126	Laridae	Larus hyperboreus	Glaucous Gull		LC
127	Laridae	Larus ichthyaetus	Pallas's Gull	II	LC
128	Laridae	Larus leucophthalmus	White-eyed Gull	I/II	NT
129	Laridae	Larus livens	Yellow-footed Gull		LC
130	Laridae	Larus maculipennis	Brown-hooded Gull		LC
131	Laridae	Larus marinus	Great Black-backed Gull		LC
132	Laridae	Larus melanocephalus	Mediterranean Gull	II	LC
133	Laridae	Larus michahellis	Yellow-legged Gull		LC
134	Laridae	Larus modestus	Grey Gull		LC
135	Laridae	Larus novaehollandiae	Silver Gull		LC
136	Laridae	Larus occidentalis	Western Gull		LC
137	Laridae	Larus pacificus	Pacific Gull		LC
138	Laridae	Larus philadelphia	Bonaparte's Gull		LC
139	Laridae	Larus pipixcan	Franklin's Gull		LC
140	Laridae	Larus relictus	Relict Gull	I	VU
141	Laridae	Larus ridibundus	Black-headed Gull		LC
142	Laridae	Larus saundersi			NA
143	Laridae	Larus schistisagus	Slaty-backed Gull		LC
144	Laridae	Larus scopulinus			NA
145	Laridae	Larus scoresbii	Dolphin Gull		LC
146	Laridae	Larus serranus	Andean Gull		LC
147	Laridae	Larus smithsonianus	Arctic Herring Gull		LC
148	Laridae	Larus thayeri	Thayer's Gull		LC
149	Laridae	Onychoprion aleuticus	Aleutian Tern		VU
150	Laridae	Onychoprion anaethetus	Bridled Tern		LC
151	Laridae	Onychoprion fuscatus	Sooty Tern		LC
152	Laridae	Onychoprion lunatus	Grey-backed Tern		LC
153	Laridae	Pagophila eburnea	Ivory Gull		NT
154	Laridae	Phaetusa simplex	Large-billed Tern		LC
155	Laridae	Procelsterna cerulea	Blue Noddy		LC
156	Laridae	Rhodostethia rosea	Ross's Gull		LC
157	Laridae	Rissa brevirostris	Red-legged Kittiwake		VU
158	Laridae	Rissa tridactyla	Black-legged Kittiwake		VU
159	Laridae	Rynchops niger	Black Skimmer		LC
160	Laridae	Saundersilarus saundersi	Saunders's Gull	I	VU
161	Laridae	Sterna dougallii	Roseate Tern	II	LC
162	Laridae	Sterna forsteri	Forster's Tern		LC
163	Laridae	Sterna hirundinacea	South American Tern		LC
164	Laridae	Sterna hirundo	Common Tern	II	LC

	Family	Scientific Name	Common Name	CMS Annex (as of Aug 7 2018)	IUCN Red List Status
165	Laridae	Sterna paradisaea	Arctic Tern	II	LC
166	Laridae	Sterna repressa	White-cheeked Tern	II	LC
167	Laridae	Sterna striata	White-fronted Tern		LC
168	Laridae	Sterna sumatrana	Black-naped Tern		LC
169	Laridae	Sterna trudeaui	Snowy-crowned Tern		LC
170	Laridae	Sterna virgata	Kerguelen Tern		NT
171	Laridae	Sterna vittata	Antarctic Tern		LC
172	Laridae	Sternula albifrons	Little Tern	II	LC
173	Laridae	Sternula antillarum	Least Tern		LC
174	Laridae	Sternula balaenarum	Damara Tern	II	VU
175	Laridae	Sternula lorata	Peruvian Tern	I	EN
176	Laridae	Sternula nereis	Fairy Tern		VU
177	Laridae	Sternula saundersi	Saunders's Tern	II	LC
178	Laridae	Thalasseus bengalensis	Lesser Crested Tern	II	LC
179	Laridae	Thalasseus bergii	Greater Crested Tern	II	LC
180	Laridae	Thalasseus bernsteini	Chinese Crested Tern	I	CR
181	Laridae	Thalasseus elegans	Elegant Tern		NT
182	Laridae	Thalasseus maximus	Royal Tern	II	LC
183	Laridae	Thalasseus sandvicensis	Sandwich Tern	II	LC
184	Laridae	Xema sabini	Sabine's Gull		LC
185	Oceanitidae	Fregetta grallaria	White-bellied Storm-petrel		LC
186	Oceanitidae	Fregetta maoriana	New Zealand Storm-petrel		CR
187	Oceanitidae	Fregetta tropica	Black-bellied Storm-petrel		LC
188	Oceanitidae	Garrodia nereis	Grey-backed Storm-petrel		LC
189	Oceanitidae	Nesofregetta fuliginosa	Polynesian Storm-petrel		EN
190	Oceanitidae	Oceanites gracilis	White-vented Storm-petrel		DD
191	Oceanitidae	Oceanites oceanicus	Wilson's Storm-petrel		LC
192	Oceanitidae	Pelagodroma marina	White-faced Storm-petrel		LC
193	Pelecanidae	Pelecanus conspicillatus	Australian Pelican		LC
194	Pelecanidae	Pelecanus erythrorhynchos	American White Pelican		LC
195	Pelecanidae	Pelecanus occidentalis	Brown Pelican		LC
196	Pelecanidae	Pelecanus onocrotalus	Great White Pelican	I/II	LC
197	Pelecanidae	Pelecanus thagus	Peruvian Pelican		NT
198	Phaethontidae	Phaethon aethereus	Red-billed Tropicbird		LC
199	Phaethontidae	Phaethon lepturus	White-tailed Tropicbird		LC
200	Phaethontidae	Phaethon rubricauda	Red-tailed Tropicbird		LC
201	Phalacrocoracidae	Microcarbo melanoleucos	Little Pied Cormorant		LC
202	Phalacrocoracidae	Phalacrocorax aristotelis	European Shag		LC
203	Phalacrocoracidae	Phalacrocorax atriceps	Imperial Shag		LC
204	Phalacrocoracidae	Phalacrocorax auritus	Double-crested Cormorant		LC
205	Phalacrocoracidae	Phalacrocorax brasilianus	Neotropical Cormorant		LC

	Family	Scientific Name	Common Name	CMS Annex (as of Aug 7 2018)	IUCN Red List Status
206	Phalacrocoracidae	Phalacrocorax capensis	Cape Cormorant		EN
207	Phalacrocoracidae	Phalacrocorax capillatus	Japanese Cormorant		LC
208	Phalacrocoracidae	Phalacrocorax carbo	Great Cormorant		LC
209	Phalacrocoracidae	Phalacrocorax magellanicus	Rock Shag		LC
210	Phalacrocoracidae	Phalacrocorax nigrogularis	Socotra Cormorant	II	VU
211	Phalacrocoracidae	Phalacrocorax pelagicus	Pelagic Cormorant		LC
212	Phalacrocoracidae	Phalacrocorax penicillatus	Brandt's Cormorant		LC
213	Phalacrocoracidae	Phalacrocorax urile	Red-faced Cormorant		LC
214	Phalacrocoracidae	Phalacrocorax verrucosus	Kerguelen Shag		LC
215	Podicipedidae	Podiceps auritus	Horned Grebe	II	VU
216	Podicipedidae	Podiceps cristatus	Great Crested Grebe		LC
217	Podicipedidae	Podiceps grisegena	Red-necked Grebe	II	LC
218	Podicipedidae	Podiceps nigricollis	Black-necked Grebe		LC
219	Procellariidae	Aphrodroma brevirostris	Kerguelen Petrel		LC
220	Procellariidae	Ardenna bulleri	Buller's Shearwater		VU
221	Procellariidae	Ardenna carneipes	Flesh-footed Shearwater		NT
222	Procellariidae	Ardenna creatopus	Pink-footed Shearwater	I	VU
223	Procellariidae	Ardenna gravis	Great Shearwater		LC
224	Procellariidae	Ardenna grisea	Sooty Shearwater		NT
225	Procellariidae	Ardenna pacifica	Wedge-tailed Shearwater		LC
226	Procellariidae	Ardenna tenuirostris	Short-tailed Shearwater		LC
227	Procellariidae	Bulweria bulwerii	Bulwer's Petrel		LC
228	Procellariidae	Bulweria fallax	Jouanin's Petrel		NT
229	Procellariidae	Calonectris borealis	Cory's Shearwater		LC
230	Procellariidae	Calonectris diomedea	Scopoli's Shearwater		LC
231	Procellariidae	Calonectris edwardsii	Cape Verde Shearwater		NT
232	Procellariidae	Calonectris leucomelas	Streaked Shearwater		NT
233	Procellariidae	Daption capense	Cape Petrel		LC
234	Procellariidae	Fulmarus glacialis	Northern Fulmar		LC
235	Procellariidae	Fulmarus glacialoides	Southern Fulmar		LC
236	Procellariidae	Halobaena caerulea	Blue Petrel		LC
237	Procellariidae	Macronectes giganteus	Southern Giant Petrel	II	LC
238	Procellariidae	Macronectes halli	Northern Giant Petrel	II	LC
239	Procellariidae	Pachyptila belcheri	Slender-billed Prion		LC
240	Procellariidae	Pachyptila crassirostris	Fulmar Prion		LC
241	Procellariidae	Pachyptila desolata	Antarctic Prion		LC
242	Procellariidae	Pachyptila macgillivrayi	MacGillivray's Prion		EN
243	Procellariidae	Pachyptila salvini	Salvin's Prion		LC
244	Procellariidae	Pachyptila turtur	Fairy Prion		LC
245	Procellariidae	Pachyptila vittata	Broad-billed Prion		LC
246	Procellariidae	Pagodroma nivea	Snow Petrel		LC

	Family	Scientific Name	Common Name	CMS Annex (as of Aug 7 2018)	IUCN Red List Status
247	Procellariidae	Pelecanoides garnotii	Peruvian Diving-petrel	I	EN
248	Procellariidae	Pelecanoides georgicus	South Georgia Diving-petrel		LC
249	Procellariidae	Pelecanoides magellani	Magellanic Diving-petrel		LC
250	Procellariidae	Pelecanoides urinatrix	Common Diving-petrel		LC
251	Procellariidae	Procellaria aequinoctialis	White-chinned Petrel	II	VU
252	Procellariidae	Procellaria cinerea	Grey Petrel	II	NT
253	Procellariidae	Procellaria conspicillata	Spectacled Petrel	II	VU
254	Procellariidae	Procellaria parkinsoni	Black Petrel	II	VU
255	Procellariidae	Procellaria westlandica	Westland Petrel	II	EN
256	Procellariidae	Pseudobulweria aterrima	Mascarene Petrel		CR
257	Procellariidae	Pseudobulweria becki	Beck's Petrel		CR
258	Procellariidae	Pseudobulweria macgillivrayi	Fiji Petrel		CR
259	Procellariidae	Pseudobulweria rostrata	Tahiti Petrel		NT
260	Procellariidae	Pterodroma alba	Phoenix Petrel		EN
261	Procellariidae	Pterodroma arminjoniana	Trindade Petrel		VU
262	Procellariidae	Pterodroma atrata	Henderson Petrel	I	EN
263	Procellariidae	Pterodroma axillaris	Chatham Petrel		VU
264	Procellariidae	Pterodroma baraui	Barau's Petrel		EN
265	Procellariidae	Pterodroma brevipes	Collared Petrel		VU
266	Procellariidae	Pterodroma cahow	Bermuda Petrel	I	EN
267	Procellariidae	Pterodroma caribbaea	Jamaican Petrel		CR
268	Procellariidae	Pterodroma cervicalis	White-necked Petrel		VU
269	Procellariidae	Pterodroma cookii	Cook's Petrel		VU
270	Procellariidae	Pterodroma defilippiana	Masatierra Petrel		VU
271	Procellariidae	Pterodroma deserta	Desertas Petrel		VU
272	Procellariidae	Pterodroma externa	Juan Fernandez Petrel		VU
273	Procellariidae	Pterodroma feae	Cape Verde Petrel		NT
274	Procellariidae	Pterodroma gouldi	Grey-faced Petrel		LC
275	Procellariidae	Pterodroma hasitata	Black-capped Petrel		EN
276	Procellariidae	Pterodroma heraldica	Herald Petrel		LC
277	Procellariidae	Pterodroma hypoleuca	Bonin Petrel		LC
278	Procellariidae	Pterodroma incerta	Atlantic Petrel		EN
279	Procellariidae	Pterodroma inexpectata	Mottled Petrel		NT
280	Procellariidae	Pterodroma lessonii	White-headed Petrel		LC
281	Procellariidae	Pterodroma leucoptera	White-winged Petrel		VU
282	Procellariidae	Pterodroma longirostris	Stejneger's Petrel		VU
283	Procellariidae	Pterodroma macroptera	Great-winged Petrel		LC
284	Procellariidae	Pterodroma madeira	Zino's Petrel		EN
285	Procellariidae	Pterodroma magentae	Magenta Petrel		CR
286	Procellariidae	Pterodroma mollis	Soft-plumaged Petrel		LC
287	Procellariidae	Pterodroma neglecta	Kermadec Petrel		LC

	Family	Scientific Name	Common Name	CMS Annex (as of Aug 7 2018)	IUCN Red List Status
288	Procellariidae	Pterodroma nigripennis	Black-winged Petrel		LC
289	Procellariidae	Pterodroma phaeopygia	Galapagos Petrel	I	CR
290	Procellariidae	Pterodroma pycrofti	Pycroft's Petrel		VU
291	Procellariidae	Pterodroma sandwichensis	Hawaiian Petrel	I	VU
292	Procellariidae	Pterodroma solandri	Providence Petrel		VU
293	Procellariidae	Pterodroma ultima	Murphy's Petrel		NT
294	Procellariidae	Puffinus assimilis	Little Shearwater		LC
295	Procellariidae	Puffinus auricularis	Townsend's Shearwater		CR
296	Procellariidae	Puffinus bailloni	Tropical Shearwater		LC
297	Procellariidae	Puffinus bannermani	Bannerman's Shearwater		EN
298	Procellariidae	Puffinus bryani	Bryan's Shearwater		CR
299	Procellariidae	Puffinus elegans	Subantarctic Shearwater		LC
300	Procellariidae	Puffinus gavia	Fluttering Shearwater		LC
301	Procellariidae	Puffinus heinrothi	Heinroth's Shearwater		VU
302	Procellariidae	Puffinus huttoni	Hutton's Shearwater		EN
303	Procellariidae	Puffinus lherminieri	Audubon's Shearwater		LC
304	Procellariidae	Puffinus mauretanicus	Balearic Shearwater	I	CR
305	Procellariidae	Puffinus nativitatis	Christmas Shearwater		LC
306	Procellariidae	Puffinus newelli	Newell's Shearwater		EN
307	Procellariidae	Puffinus opisthomelas	Black-vented Shearwater		NT
308	Procellariidae	Puffinus persicus	Persian Shearwater		LC
309	Procellariidae	Puffinus puffinus	Manx Shearwater		LC
310	Procellariidae	Puffinus subalaris	Galapagos Shearwater		LC
311	Procellariidae	Puffinus yelkouan	Yelkouan Shearwater		VU
312	Procellariidae	Thalassoica antarctica	Antarctic Petrel		LC
313	Scolopacidae	Phalaropus fulicarius	Red Phalarope	II	LC
314	Scolopacidae	Phalaropus lobatus	Red-necked Phalarope	II	LC
315	Spheniscidae	Aptenodytes forsteri	Emperor Penguin		NT
316	Spheniscidae	Aptenodytes patagonicus	King Penguin		LC
317	Spheniscidae	Eudyptes chrysocome	Southern Rockhopper Penguin		VU
318	Spheniscidae	Eudyptes chrysolophus	Macaroni Penguin		VU
319	Spheniscidae	Eudyptes moseleyi	Northern Rockhopper Penguin		EN
320	Spheniscidae	Eudyptes pachyrhynchus	Fiordland Penguin		VU
321	Spheniscidae	Eudyptes robustus	Snares Penguin		VU
322	Spheniscidae	Eudyptes sclateri	Erect-crested Penguin		EN
323	Spheniscidae	Eudyptula minor	Little Penguin		LC
324	Spheniscidae	Pygoscelis adeliae	Adelie Penguin		LC
325	Spheniscidae	Pygoscelis antarcticus	Chinstrap Penguin		LC
326	Spheniscidae	Pygoscelis papua	Gentoo Penguin		LC
327	Spheniscidae	Spheniscus demersus	African Penguin	II	EN
328	Spheniscidae	Spheniscus humboldti	Humboldt Penguin	I	VU

	Family	Scientific Name	Common Name	CMS Annex (as of Aug 7 2018)	IUCN Red List Status
329	Spheniscidae	Spheniscus magellanicus	Magellanic Penguin		NT
330	Stercorariidae	Catharacta antarctica	Brown Skua		LC
331	Stercorariidae	Catharacta chilensis	Chilean Skua		LC
332	Stercorariidae	Catharacta maccormicki	South Polar Skua		LC
333	Stercorariidae	Catharacta skua	Great Skua		LC
334	Stercorariidae	Stercorarius longicaudus	Long-tailed Jaeger		LC
335	Stercorariidae	Stercorarius parasiticus	Arctic Jaeger		LC
336	Stercorariidae	Stercorarius pomarinus	Pomarine Jaeger		LC
337	Sulidae	Morus bassanus	Northern Gannet		LC
338	Sulidae	Morus capensis	Cape Gannet		EN
339	Sulidae	Morus serrator	Australasian Gannet		LC
340	Sulidae	Papasula abbotti	Abbott's Booby		EN
341	Sulidae	Sula dactylatra	Masked Booby		LC
342	Sulidae	Sula granti	Nazca Booby		LC
343	Sulidae	Sula leucogaster	Brown Booby		LC
344	Sulidae	Sula nebouxii	Blue-footed Booby		LC
345	Sulidae	Sula sula	Red-footed Booby		LC
346	Sulidae	Sula variegata	Peruvian Booby		LC

	Family	Scientific Name	Common Name	CMS Annex (as of Aug 7 2018)	IUCN Red List Status
1	Cheloniidae	Caretta caretta	Loggerhead Turtle	I/II	VU
2	Cheloniidae	Chelonia mydas	Green Turtle	I/II	EN
3	Cheloniidae	Eretmochelys imbricata	Hawksbill Turtle	I/II	CR
4	Cheloniidae	Lepidochelys kempii	Kemp's Ridley	I/II	CR
5	Cheloniidae	Lepidochelys olivacea	Olive Ridley	I/II	VU
6	Cheloniidae	Natator depressus	Flatback	II	DD
7	Dermochelyidae	Dermochelys coriacea	Leatherback	I/II	VU

	Family	Scientific Name	Common Name	CMS Annex (as of Aug 7 2018)	IUCN Red List Status
1	Acipenseridae	Acipenser brevirostrum	Shortnose Sturgeon		VU
2	Acipenseridae	Acipenser medirostris	Green Sturgeon	II	NT
3	Acipenseridae	Acipenser oxyrinchus	Gulf Sturgeon		NT
4	Acipenseridae	Acipenser sinensis	Chinese Sturgeon	II	CR
5	Acipenseridae	Acipenser stellatus	Stellate Sturgeon	II	CR
6	Acipenseridae	Acipenser sturio	Atlantic Sturgeon	I/II	CR
7	Aetobatidae	Aetobatus flagellum	Longhead Eagle Ray		EN
8	Aetobatidae	Aetobatus narinari	Spotted Eagle Ray		NT
9	Alopiidae	Alopias pelagicus	Pelagic Thresher	II	VU
10	Alopiidae	Alopias superciliosus	Bigeye Thresher Shark	II	VU
11	Alopiidae	Alopias vulpinus	Common Thresher Shark	II	VU
12	Anguillidae	Anguilla anguilla	European Eel	II	CR
13	Anguillidae	Anguilla bengalensis	Indian Mottled Eel		NT
14	Anguillidae	Anguilla bicolor	Shortfin Eel		NT
15	Anguillidae	Anguilla marmorata	Marbled Eel		LC
16	Anguillidae	Anguilla nebulosa	Indian Mottled Eel		NA
17	Belonidae	Platybelone argalus	Keeltail Needlefish		LC
18	Belonidae	Strongylura exilis	Californian Needlefish		LC
19	Belonidae	Strongylura marina	Atlantic Needlefish		LC
20	Belonidae	Strongylura scapularis	Shoulderspot needlefish		LC
21	Belonidae	Tylosurus pacificus	Pacific agujon needlefish		LC
22	Bramidae	Brama dussumieri	Lesser Bream		LC
23	Bramidae	Pteraclis aesticola	Pacific Fanfish		LC
24	Carangidae	Alectis ciliaris	African Pompano		LC
25	Carangidae	Alepes vari	Herring Scad		LC
26	Carangidae	Carangoides otrynter	Threadfin Jack		LC
27	Carangidae	Caranx caballus	Green Jack		LC
28	Carangidae	Caranx caninus	Pacific Crevalle Jack		LC
29	Carangidae	Caranx crysos	Blue Runner		LC
30	Carangidae	Caranx sexfasciatus	Bigeye Trevally		LC
31	Carangidae	Caranx vinctus	Cocinero		LC
32	Carangidae	Chloroscombrus orqueta	Pacific Bumper		LC
33	Carangidae	Decapterus russelli	Indian Scad		LC
34	Carangidae	Hemicaranx leucurus	Yellowfin Jack		LC
35	Carangidae	Hemicaranx zelotes	Blackfin Jack		LC
36	Carangidae	Oligoplites altus	Longjaw Leatherjacket		LC
37	Carangidae	Oligoplites refulgens	Shortjaw Leatherjack		LC
38	Carangidae	Selene brevoortii	Mexican Lookdown		LC
39	Carangidae	Selene orstedii	Mexican Moonfish		LC
40	Carangidae	Selene peruviana	Pacific Moonfish		LC
41	Carangidae	Seriola peruana	Darkfin amberjack		LC
42	Carangidae	Trachinotus goodei	Palometa		LC

	Family	Scientific Name	Common Name	CMS Annex (as of Aug 7 2018)	IUCN Red List Status
43	Carangidae	Trachinotus kennedyi	Blackblotch Pompano		LC
44	Carangidae	Trachinotus paitensis	Paloma Pompano		LC
45	Carangidae	Trachinotus rhodopus	Gafftopsail Pompano		LC
46	Carangidae	Trachinotus stilbe	Steel Pompano		LC
47	Carangidae	Trachurus murphyi	Chilean Jack Mackerel		DD
48	Carangidae	Trachurus symmetricus	Pacific Jack Mackerel		LC
49	Carcharhinidae	Carcharhinus acronotus	Blacknose Shark		NT
50	Carcharhinidae	Carcharhinus albimarginatus	Silvertip Shark		VU
51	Carcharhinidae	Carcharhinus altimus	Bignose Shark		DD
52	Carcharhinidae	Carcharhinus amblyrhynchoides	Graceful Shark		NT
53	Carcharhinidae	Carcharhinus amblyrhynchos	Grey Reef Shark		NT
54	Carcharhinidae	Carcharhinus amboinensis	Pigeye Shark		DD
55	Carcharhinidae	Carcharhinus borneensis	Borneo Shark		EN
56	Carcharhinidae	Carcharhinus brachyurus	Copper Shark		NT
57	Carcharhinidae	Carcharhinus brevipinna	Spinner Shark		NT
58	Carcharhinidae	Carcharhinus cautus	Nervous Shark		DD
59	Carcharhinidae	Carcharhinus dussumieri	Widemouth Blackspot Shark		NT
60	Carcharhinidae	Carcharhinus falciformis	Silky Shark	II	VU
61	Carcharhinidae	Carcharhinus fitzroyensis	Creek Whaler		LC
62	Carcharhinidae	Carcharhinus galapagensis	Galapagos Shark		NT
63	Carcharhinidae	Carcharhinus hemiodon	Pondicherry Shark		CR
64	Carcharhinidae	Carcharhinus isodon	Finetooth Shark		LC
65	Carcharhinidae	Carcharhinus leiodon	Smoothtooth Blacktip Shark		EN
66	Carcharhinidae	Carcharhinus leucas	Bull Shark		NT
67	Carcharhinidae	Carcharhinus limbatus	Blacktip Shark		NT
68	Carcharhinidae	Carcharhinus longimanus	Oceanic Whitetip Shark		VU
69	Carcharhinidae	Carcharhinus macloti	Hardnose Shark		NT
70	Carcharhinidae	Carcharhinus melanopterus	Blacktip Reef Shark		NT
71	Carcharhinidae	Carcharhinus obscurus	Dusky Shark	II	VU
72	Carcharhinidae	Carcharhinus perezi	Caribbean Reef Shark		NT
73	Carcharhinidae	Carcharhinus plumbeus	Sandbar Shark		VU
74	Carcharhinidae	Carcharhinus porosus	Smalltail Shark		DD
75	Carcharhinidae	Carcharhinus sealei	Blackspot Shark		NT
76	Carcharhinidae	Carcharhinus signatus	Night Shark		VU
77	Carcharhinidae	Carcharhinus sorrah	Spottail Shark		NT
78	Carcharhinidae	Carcharhinus tilstoni	Australian Blacktip Shark		LC
79	Carcharhinidae	Galeocerdo cuvier	Tiger Shark		NT
80	Carcharhinidae	Glyphis gangeticus	Ganges Shark		CR
81	Carcharhinidae	Glyphis garricki	New Guinea River Shark		CR
82	Carcharhinidae	Glyphis glyphis	Speartooth Shark		EN
83	Carcharhinidae	Glyphis siamensis	Irrawaddy River Shark		CR
84	Carcharhinidae	Isogomphodon oxyrhynchus	Daggernose Shark		CR

	Family	Scientific Name	Common Name	CMS Annex (as of Aug 7 2018)	IUCN Red List Status
85	Carcharhinidae	Lamiopsis temmincki	Broadfin Shark		NA
86	Carcharhinidae	Loxodon macrorhinus	Sliteye Shark		LC
87	Carcharhinidae	Nasolamia velox	Whitenose Shark		DD
88	Carcharhinidae	Negaprion acutidens	Sharptooth Lemon Shark		VU
89	Carcharhinidae	Negaprion brevirostris	Lemon Shark		NT
90	Carcharhinidae	Prionace glauca	Blue Shark	II	NT
91	Carcharhinidae	Rhizoprionodon acutus	Milk Shark		LC
92	Carcharhinidae	Rhizoprionodon lalandii	Brazilian Sharpnose Shark		DD
93	Carcharhinidae	Rhizoprionodon longurio	Pacific Sharpnose Shark		DD
94	Carcharhinidae	Rhizoprionodon oligolinx	Grey Sharpnose Shark		LC
95	Carcharhinidae	Rhizoprionodon porosus	Caribbean Sharpnose Shark		LC
96	Carcharhinidae	Rhizoprionodon taylori	Australian Sharpnose Shark		LC
97	Carcharhinidae	Rhizoprionodon terraenovae	Atlantic Sharpnose Shark		LC
98	Carcharhinidae	Scoliodon laticaudus	Spadenose Shark		NT
99	Carcharhinidae	Triaenodon obesus	Whitetip Reef Shark		NT
100	Cetorhinidae	Cetorhinus maximus	Basking Shark	I/II	VU
101	Clupeidae	Alosa aestivalis	Blueback Herring		VU
102	Clupeidae	Alosa alabamae	Alabama Shad		DD
103	Clupeidae	Alosa caspia			LC
104	Clupeidae	Alosa immaculata			VU
105	Clupeidae	Alosa mediocris	Hickory Shad		LC
106	Clupeidae	Alosa pseudoharengus	Alewife		LC
107	Clupeidae	Alosa sapidissima	American Shad		LC
108	Clupeidae	Anodontostoma selangkat	Indonesian Gizzard Shad		DD
109	Clupeidae	Anodontostoma thailandiae	Thai Gizzard Shad		LC
110	Clupeidae	Clupea harengus	Atlantic Herring		LC
111	Clupeidae	Ethmalosa fimbriata	Bonga shad		LC
112	Clupeidae	Etrumeus whiteheadi	Whitehead's Round Herring		LC
113	Clupeidae	Gonialosa modesta	Burmese River Gizzard Shad		LC
114	Clupeidae	Gonialosa whiteheadi	Southern Burmese Gizzard Shad		DD
115	Clupeidae	Harengula thrissina	Pacific Flatiron Herring		LC
116	Clupeidae	Jenkinsia lamprotaenia	Dwarf Round Herring		LC
117	Clupeidae	Jenkinsia majua	Little-eye Round Herring		LC
118	Clupeidae	Jenkinsia parvula	Venezuelan Herring		DD
119	Clupeidae	Lile gracilis	Graceful Piquitinga		LC
120	Clupeidae	Lile nigrofasciata	Blackstripe Herring		LC
121	Clupeidae	Lile piquitinga	Atlantic Piquitinga		LC
122	Clupeidae	Lile stolifera	Pacific Piquitinga		LC
123	Clupeidae	Minyclupeoides dentibranchialus			LC
124	Clupeidae	Nematalosa galatheae	Galathea Gizzard Shad		LC
125	Clupeidae	Nematalosa nasus	Bloch's Gizzard Shad		LC
126	Clupeidae	Opisthonema berlangai	Galapv°gos Thread Herring		VU

	Family	Scientific Name	Common Name	CMS Annex (as of Aug 7 2018)	IUCN Red List Status
127	Clupeidae	Opisthonema bulleri	Slender thread herring		LC
128	Clupeidae	Opisthonema libertate	Deep-bodied Pacific thread herring		LC
129	Clupeidae	Opisthonema medirastre	Middling thread herring		LC
130	Clupeidae	Pellonula vorax	Bigtoothed Pellonula		LC
131	Clupeidae	Pliosteostoma lutipinnis	Yellowfin Herring		LC
132	Clupeidae	Sardinella albella	White Sardinella		LC
133	Clupeidae	Sardinella fijiense	Fiji Sardinella		LC
134	Clupeidae	Sardinella longiceps	Indian Oil Sardine		LC
135	Clupeidae	Spratelloides gracilis	Blue Sprat		LC
136	Coryphaenidae	Coryphaena equiselis	Pompano Dolphinfish		LC
137	Coryphaenidae	Coryphaena hippurus	Common Dolphinfish		LC
138	Dalatiidae	Dalatias licha	Kitefin Shark		NT
139	Dalatiidae	Euprotomicroides zantedeschia	Taillight Shark		DD
140	Dalatiidae	Euprotomicrus bispinatus	Pygmy Shark		LC
141	Dalatiidae	Heteroscymnoides marleyi	Longnose Pygmy Shark		LC
142	Dalatiidae	Isistius brasiliensis	Cookie-cutter Shark		LC
143	Dalatiidae	Isistius labialis	South China Cookiecutter Shark		DD
144	Dalatiidae	Isistius plutodus	Largetooth Cookiecutter Shark		LC
145	Dalatiidae	Mollisquama parini	Pocket Shark		DD
146	Dalatiidae	Squaliolus aliae	Smalleye Pygmy Shark		LC
147	Dalatiidae	Squaliolus laticaudus	Spined Pygmy Shark		LC
148	Dasyatidae	Dasyatis centroura	Roughtail Stingray		NA
149	Dasyatidae	Dasyatis colarensis	Colares Stringray		NA
150	Dasyatidae	Dasyatis geijskesi	Sharpsnout Stingray		NA
151	Dasyatidae	Dasyatis sabina	Atlantic Stingray		NA
152	Dasyatidae	Himantura fai	Pink Whipray		NA
153	Dasyatidae	Himantura imbricata	Scaly Whipray		NA
154	Dasyatidae	Himantura marginata	Blackedge Whipray		DD
155	Dasyatidae	Himantura uarnacoides	Bleeker's Whipray		NA
156	Dasyatidae	Himantura uarnak	Reticulate Whipray		VU
157	Dasyatidae	Pastinachus sephen	Cowtail Ray		NT
158	Dasyatidae	Pteroplatytrygon violacea	Pelagic Stingray		LC
159	Echinorhinidae	Echinorhinus brucus	Bramble Shark		DD
160	Echinorhinidae	Echinorhinus cookei	Prickly Shark		NT
161	Engraulidae	Anchoa analis	Longfin Pacific Anchovy		DD
162	Engraulidae	Anchoa argentivittata	Silverstripe Anchovy		LC
163	Engraulidae	Anchoa chamensis	Chame Point anchovy		VU
164	Engraulidae	Anchoa choerostoma	Bermuda Anchovy		EN
165	Engraulidae	Anchoa curta	Short Anchovy		LC
166	Engraulidae	Anchoa delicatissima	Slough Anchovy		LC
167	Engraulidae	Anchoa eigenmannia	Eigenmann's Anchovy		LC
168	Engraulidae	Anchoa exigua	Slender Anchovy		LC

	Family	Scientific Name	Common Name	CMS Annex (as of Aug 7 2018)	IUCN Red List Status
169	Engraulidae	Anchoa helleri	Heller's Anchovy		LC
170	Engraulidae	Anchoa ischana	Sharpnose Anchovy		LC
171	Engraulidae	Anchoa lucida	Bright Anchovy		LC
172	Engraulidae	Anchoa mitchilli	Bay Anchovy		LC
173	Engraulidae	Anchoa mundeola	False Panama Anchovy		LC
174	Engraulidae	Anchoa mundeoloides	Northern Gulf Anchovy		LC
175	Engraulidae	Anchoa nasus	Longnose Anchovy		LC
176	Engraulidae	Anchoa panamensis	Panama Anchovy		LC
177	Engraulidae	Anchoa scofieldi	Scofield's Anchovy		LC
178	Engraulidae	Anchoa starksi	Starks's Anchovy		LC
179	Engraulidae	Anchoa walkeri	Walker's Anchovy		LC
180	Engraulidae	Anchovia macrolepidota	Bigscale Anchovy		LC
181	Engraulidae	Anchoviella balboae	Balboa Anchovy		DD
182	Engraulidae	Anchoviella brevirostris	Snubnose Anchovy		LC
183	Engraulidae	Cetengraulis mysticetus	Pacific Anchoveta		LC
184	Engraulidae	Coilia grayii	Grey's Grenadier Anchovy		LC
185	Engraulidae	Coilia lindmani	Lindman's Grenadier Anchovy		LC
186	Engraulidae	Coilia neglecta	Neglected Grenadier Anchovy		LC
187	Engraulidae	Engraulis mordax	California Anchovy		LC
188	Engraulidae	Engraulis ringens	Peruvian Anchoveta		LC
189	Engraulidae	Lycengraulis poeyi	Pacific Sabretooth Anchovy		LC
190	Engraulidae	Stolephorus ronquilloi	Ronquillo's Anchovy		VU
191	Engraulidae	Thryssa gautamiensis	Gautama Thryssa		DD
192	Engraulidae	Thryssa mystax	Moustached Thryssa		LC
193	Engraulidae	Thryssa scratchleyi	Freshwater Anchovy		DD
194	Exocoetidae	Cheilopogon dorsomacula	Backspot Flying Fish		LC
195	Exocoetidae	Cheilopogon papilio	Butterfly flyingfish		LC
196	Exocoetidae	Cheilopogon pinnatibarbatus	Bennett's Flyingfish		LC
197	Exocoetidae	Cheilopogon rapanouiensis	Easter Island Flyingfish		LC
198	Exocoetidae	Cheilopogon xenopterus	Whitetip flyingfish		LC
199	Exocoetidae	Cypselurus callopterus	Ornamented flyingfish		LC
200	Exocoetidae	Exocoetus peruvianus	Peruvian Flyingfish		DD
201	Exocoetidae	Fodiator rostratus	Sharpchin flyingfish		LC
202	Exocoetidae	Hirundichthys marginatus	Banded Flyingfish		LC
203	Exocoetidae	Hirundichthys rondeletii	Atlantic Black Wing Flyingfish		LC
204	Exocoetidae	Prognichthys sealei	Sailor flyingfish		LC
205	Exocoetidae	Prognichthys tringa	Tringa flyingfish		LC
206	Ginglymostomatidae	Nebrius ferrugineus	Tawny Nurse Shark		VU
207	Hemiramphidae	Hemiramphus saltator	Jumping halfbeak		LC
208	Hemiramphidae	Hyporhamphus gilli	Choelo halfbeak		LC
209	Hemiramphidae	Hyporhamphus limbatus	Congaturi Halfbeak		LC
210	Hemiramphidae	Hyporhamphus naos	Pacific silverstripe halfbeak		LC

	Family	Scientific Name	Common Name	CMS Annex (as of Aug 7 2018)	IUCN Red List Status
211	Hemiramphidae	Hyporhamphus roberti	Central American Halfbeak		LC
212	Hemiramphidae	Hyporhamphus rosae	California Halfbeak		DD
213	Hemiramphidae	Hyporhamphus snyderi	Skipper halfbeak		LC
214	Hemiramphidae	Zenarchopterus caudovittatus			DD
215	Hemiramphidae	Zenarchopterus dispar	Feathered River-garfish		LC
216	Hemiramphidae	Zenarchopterus gilli			LC
217	Hemiramphidae	Zenarchopterus pappenheimi	Bangkok Halfbeak		DD
218	Hexanchidae	Hexanchus griseus	Bluntnose Sixgill Shark		NT
219	Hexanchidae	Notorynchus cepedianus	Broadnose Sevengill Shark		DD
220	Istiophoridae	Istiompax indica	Black Marlin		DD
221	Istiophoridae	Istiophorus albicans	Atlantic Sailfish		NA
222	Istiophoridae	Istiophorus platypterus	Sailfish		LC
223	Istiophoridae	Kajikia albida	White Marlin		VU
224	Istiophoridae	Kajikia audax	Striped Marlin		NT
225	Istiophoridae	Makaira nigricans	Blue Marlin		VU
226	Istiophoridae	Tetrapturus angustirostris	Shortbill Spearfish		DD
227	Istiophoridae	Tetrapturus belone	Mediterranean Shortbill Spearfish		LC
228	Istiophoridae	Tetrapturus georgii	Roundscale Spearfish		DD
229	Istiophoridae	Tetrapturus pfluegeri	Longbill Spearfish		LC
230	Lamnidae	Carcharodon carcharias	Great White Shark	I/II	VU
231	Lamnidae	Isurus oxyrinchus	Shortfin Mako	II	VU
232	Lamnidae	Isurus paucus	Longfin Mako	II	VU
233	Lamnidae	Lamna ditropis	Salmon Shark		LC
234	Lamnidae	Lamna nasus	Porbeagle	II	VU
235	Lutjanidae	Apsilus dentatus	Black Snapper		LC
236	Lutjanidae	Etelis carbunculus	Ruby Snapper		LC
237	Lutjanidae	Hoplopagrus guentherii	Mexican barred snapper		LC
238	Lutjanidae	Lutjanus analis	Mutton Snapper		NT
239	Lutjanidae	Lutjanus aratus	Vivaneau radis		LC
240	Lutjanidae	Lutjanus argentiventris	Amarillo snapper		LC
241	Lutjanidae	Lutjanus colorado	Vivaneau amarante		LC
242	Lutjanidae	Lutjanus cyanopterus	Cubera Snapper		VU
243	Lutjanidae	Lutjanus decussatus	Crossbanded Snapper		LC
244	Lutjanidae	Lutjanus guttatus	Spotted rose snapper		LC
245	Lutjanidae	Lutjanus inermis	Golden snapper		LC
246	Lutjanidae	Lutjanus jordani	Whipper snapper		LC
247	Lutjanidae	Lutjanus notatus	Bluestriped Snapper		LC
248	Lutjanidae	Lutjanus novemfasciatus	Pacific cubera snapper		LC
249	Lutjanidae	Lutjanus peru	Pacific red snapper		LC
250	Lutjanidae	Lutjanus viridis	Blue and gold snapper		LC
251	Lutjanidae	Paracaesio caerulea	Japanese Snapper		DD
252	Lutjanidae	Pristipomoides freemani	Slender Wenchman		LC

	Family	Scientific Name	Common Name	CMS Annex (as of Aug 7 2018)	IUCN Red List Status
253	Megachasmidae	Megachasma pelagios	Megamouth Shark		LC
254	Mitsukurinidae	Mitsukurina owstoni	Goblin Shark		LC
255	Mobulidae	Mobula alfredi	Reef Manta Ray		VU
256	Mobulidae	Mobula birostris	Giant Manta Ray		VU
257	Mobulidae	Mobula eregoodoo	Longhorned Pygmy Devil Ray	I/II	NT
258	Mobulidae	Mobula hypostoma	Atlantic Devilray	I/II	DD
259	Mobulidae	Mobula kuhlii	Shortfin Devil Ray	I/II	DD
260	Mobulidae	Mobula mobular	Giant Devil Ray	I/II	EN
261	Mobulidae	Mobula munkiana	Smoothtail Mobula	I/II	NT
262	Mobulidae	Mobula rochebrunei	Lesser Guinean Devil Ray	I/II	VU
263	Mobulidae	Mobula tarapacana	Sicklefin Devil Ray	I/II	VU
264	Mobulidae	Mobula thurstoni	Bentfin Devil Ray	I/II	NT
265	Myliobatidae	Aetomylaeus maculatus	Mottled Eagle Ray		EN
266	Myliobatidae	Aetomylaeus nichofii	Banded Eagle Ray		VU
267	Myliobatidae	Aetomylaeus vespertilio	Ornate Eagle Ray		EN
268	Myliobatidae	Myliobatis aquila	Common Eagle Ray		DD
269	Myliobatidae	Myliobatis australis	Southern Eagle Ray		NA
270	Myliobatidae	Myliobatis californicus	Bat Ray		LC
271	Myliobatidae	Myliobatis chilensis	Chilean Eagle Ray		DD
272	Myliobatidae	Myliobatis freminvillii	Bullnose Ray		DD
273	Myliobatidae	Myliobatis goodei	Southern Eagle Ray		DD
274	Myliobatidae	Myliobatis hamlyni	Purple Eagle Ray		NT
275	Myliobatidae	Myliobatis longirostris	Longnose Eagle Ray		NT
276	Myliobatidae	Myliobatis peruvianus	Peruvian Eagle Ray		DD
277	Myliobatidae	Myliobatis tenuicaudatus	Southern Eagle Ray		LC
278	Myliobatidae	Myliobatis tobijei	Japanese Eagle Ray		DD
279	Myliobatidae	Pteromylaeus asperrimus	Roughskin Bullray		NA
280	Myliobatidae	Pteromylaeus bovinus	Bullray		NA
281	Odontaspididae	Carcharias taurus	Sand Tiger Shark		VU
282	Odontaspididae	Odontaspis ferox	Smalltooth Sand Tiger		VU
283	Odontaspididae	Odontaspis noronhai	Bigeye Sand Tiger		DD
284	Potamotrygonidae	Potamotrygon constellata	Thorny River Stingray		DD
285	Potamotrygonidae	Potamotrygon histrix	Porcupine River Stingray		DD
286	Potamotrygonidae	Potamotrygon motoro	Ocellate River Stingray		DD
287	Potamotrygonidae	Potamotrygon scobina	Raspy River Stingray		DD
288	Pristidae	Anoxypristis cuspidata	Narrow Sawfish	I/II	EN
289	Pristidae	Pristis pectinata	Smalltooth Sawfish		CR
290	Pristidae	Pristis pristis	Largetooth Sawfish	I/II	CR
291	Pristigasteridae	Chirocentrodon bleekerianus	Dogtooth Herring		LC
292	Pristigasteridae	Ilisha fuerthii	Hatchet Herring		LC
293	Pristigasteridae	Neoopisthopterus tropicus	Tropical longfin herring		LC
294	Pristigasteridae	Odontognathus panamensis	Panama longfin herring		LC

	Family	Scientific Name	Common Name	CMS Annex (as of Aug 7 2018)	IUCN Red List Status
295	Pristigasteridae	Opisthopterus dovii	Dove's Longfin Herring		LC
296	Pristigasteridae	Opisthopterus effulgens	Vaqueira longfin herring		VU
297	Pristigasteridae	Opisthopterus equatorialis	Equatorial longfin herring		LC
298	Pristigasteridae	Opisthopterus macrops	Bigeyed longfin herring		LC
299	Pristigasteridae	Raconda russeliana	Raconda		LC
300	Pristiophoridae	Pliotrema warreni	Sixgill Sawshark		NT
301	Pseudocarchariidae	Pseudocarcharias kamoharai	Crocodile Shark		NT
302	Rajidae	Amblyraja radiata	Thorny Skate		VU
303	Rajidae	Malacoraja senta	Smooth Skate		EN
304	Rajidae	Raja binoculata	Big Skate		NA
305	Rajidae	Raja pulchra	Mottled Skate		NA
306	Rajidae	Raja straeleni	Biscuit Skate		DD
307	Rhincodontidae	Rhincodon typus	Whale Shark	I/II	EN
308	Rhinidae	Rhynchobatus djiddensis	Whitespotted Wedgefish		VU
309	Rhinidae	Rhynchobatus luebberti	African Wedgefish		EN
310	Rhinobatidae	Rhinobatos annandalei	Bengal Guitarfish		DD
311	Rhinobatidae	Rhinobatos annulatus	Lesser Guitarfish		NA
312	Rhinobatidae	Rhinobatos horkelii	Brazilian Guitarfish		NA
313	Rhinobatidae	Rhinobatos lionotus	Smoothback Guitarfish		DD
314	Rhinopteridae	Rhinoptera bonasus	Cownose Ray		NT
315	Rhinopteridae	Rhinoptera brasiliensis	Brazilian Cownose Ray		EN
316	Rhinopteridae	Rhinoptera javanica	Javanese Cownose Ray		VU
317	Rhinopteridae	Rhinoptera neglecta	Australian Cownose Ray		DD
318	Rhinopteridae	Rhinoptera steindachneri	Pacific Cownose Ray		NT
319	Salmonidae	Coregonus artedi	Cisco		LR/lc
320	Salmonidae	Coregonus huntsmani	Atlantic Whitefish		CR
321	Salmonidae	Coregonus subautumnalis			VU
322	Salmonidae	Coregonus widegreni			DD
323	Salmonidae	Hucho perryi	Sakhalin Taimen		CR
324	Salmonidae	Oncorhynchus ishikawai	Satsukimasu Salmon		NA
325	Salmonidae	Oncorhynchus nerka	Sockeye Salmon		LC
326	Salmonidae	Salmo salar	Atlantic Salmon		LR/lc
327	Salmonidae	Salvelinus confluentus	Bull Trout		VU
328	Scomberesocidae	Cololabis adocetus			NA
329	Scomberesocidae	Cololabis saira			NA
330	Scomberesocidae	Scomberesox saurus	Atlantic Saury		LC
331	Scombridae	Acanthocybium solandri	Wahoo		LC
332	Scombridae	Allothunnus fallai	Slender Tuna		LC
333	Scombridae	Auxis rochei	Bullet Tuna		LC
334	Scombridae	Auxis thazard	Frigate Tuna		LC
335	Scombridae	Cybiosarda elegans	Leaping Bonito		LC
336	Scombridae	Euthynnus affinis	Kawakawa		LC

	Family	Scientific Name	Common Name	CMS Annex (as of Aug 7 2018)	IUCN Red List Status
337	Scombridae	Euthynnus alletteratus	Little Tunny		LC
338	Scombridae	Euthynnus lineatus	Black Skipjack		LC
339	Scombridae	Gasterochisma melampus	Butterfly Mackerel		LC
340	Scombridae	Grammatorcynus bicarinatus	Shark Mackerel		LC
341	Scombridae	Grammatorcynus bilineatus	Double-lined Mackerel		LC
342	Scombridae	Gymnosarda unicolor	Dogtooth Tuna		LC
343	Scombridae	Katsuwonus pelamis	Skipjack Tuna		LC
344	Scombridae	Orcynopsis unicolor	Plain Bonito		LC
345	Scombridae	Rastrelliger brachysoma	Short Mackerel		DD
346	Scombridae	Rastrelliger faughni	Island Mackerel		DD
347	Scombridae	Rastrelliger kanagurta	Indian Mackerel		DD
348	Scombridae	Sarda australis	Australian Bonito		LC
349	Scombridae	Sarda chiliensis	Pacific Bonito		LC
350	Scombridae	Sarda orientalis	Oriental Bonito		LC
351	Scombridae	Sarda sarda	Atlantic Bonito		LC
352	Scombridae	Scomber australasicus	Blue Mackerel		LC
353	Scombridae	Scomber colias	Atlantic Chub Mackerel		LC
354	Scombridae	Scomber japonicus	Pacific Chub Mackerel		LC
355	Scombridae	Scomber scombrus	Atlantic Mackerel		LC
356	Scombridae	Scomberomorus brasiliensis	Serra Spanish Mackerel		LC
357	Scombridae	Scomberomorus cavalla	King Mackerel		LC
358	Scombridae	Scomberomorus commerson	Narrow-barred Spanish Mackerel		NT
359	Scombridae	Scomberomorus concolor	Monterrey Spanish Mackerel		VU
360	Scombridae	Scomberomorus guttatus	Indo-Pacific King Mackerel		DD
361	Scombridae	Scomberomorus koreanus	Korean Seerfish		LC
362	Scombridae	Scomberomorus lineolatus	Streaked Seerfish		LC
363	Scombridae	Scomberomorus maculatus	Atlantic Spanish Mackerel		LC
364	Scombridae	Scomberomorus multiradiatus	Papuan Seerfish		LC
365	Scombridae	Scomberomorus munroi	Spotted Mackerel		NT
366	Scombridae	Scomberomorus niphonius	Japanese Seerfish		DD
367	Scombridae	Scomberomorus plurilineatus	Queen Mackerel		DD
368	Scombridae	Scomberomorus queenslandicus	School Mackerel		LC
369	Scombridae	Scomberomorus regalis	Cero		LC
370	Scombridae	Scomberomorus semifasciatus	Broad-barred Mackerel		LC
371	Scombridae	Scomberomorus sierra	Pacific Sierra		LC
372	Scombridae	Scomberomorus sinensis	Chinese Seerfish		DD
373	Scombridae	Scomberomorus tritor	West African Spanish Mackerel		LC
374	Scombridae	Thunnus alalunga	Albacore Tuna		NT
375	Scombridae	Thunnus albacares	Yellowfin Tuna		NT
376	Scombridae	Thunnus atlanticus	Blackfin Tuna		LC
377	Scombridae	Thunnus maccoyii	Southern Bluefin Tuna		CR
378	Scombridae	Thunnus obesus	Bigeye Tuna		VU

	Family	Scientific Name	Common Name	CMS Annex (as of Aug 7 2018)	IUCN Red List Status
379	Scombridae	Thunnus orientalis	Pacific Bluefin Tuna		VU
380	Scombridae	Thunnus thynnus	Atlantic Bluefin Tuna		EN
381	Scombridae	Thunnus tonggol	Longtail Tuna		DD
382	Somniosidae	Centroscymnus coelolepis	Portuguese Dogfish		NT
383	Somniosidae	Centroscymnus owstonii	Roughskin Dogfish		LC
384	Somniosidae	Centroselachus crepidater	Longnose Velvet Dogfish		LC
385	Somniosidae	Proscymnodon macracanthus	Largespine Velvet Dogfish		DD
386	Somniosidae	Proscymnodon plunketi	Plunket's Dogfish		NA
387	Somniosidae	Scymnodalatias albicauda	Whitetail Dogfish		DD
388	Somniosidae	Scymnodalatias garricki	Azores Dogfish		DD
389	Somniosidae	Scymnodalatias oligodon	Sparsetooth Dogfish		DD
390	Somniosidae	Scymnodalatias sherwoodi	Sherwood's Dogfish		DD
391	Somniosidae	Scymnodon ringens	Knifetooth Dogfish		DD
392	Somniosidae	Somniosus antarcticus	Southern Sleeper Shark		DD
393	Somniosidae	Somniosus longus	Frog Shark		DD
394	Somniosidae	Somniosus microcephalus	Greenland Shark		NT
395	Somniosidae	Somniosus pacificus	Pacific Sleeper Shark		DD
396	Somniosidae	Somniosus rostratus	Little Sleeper Shark		DD
397	Somniosidae	Zameus ichiharai	Japanese Velvet Dogfish		DD
398	Somniosidae	Zameus squamulosus	Velvet Dogfish		DD
399	Sphyrnidae	Eusphyra blochii	Winghead Shark		EN
400	Sphyrnidae	Sphyrna corona	Scalloped Bonnethead		NT
401	Sphyrnidae	Sphyrna lewini	Scalloped Hammerhead	II	EN
402	Sphyrnidae	Sphyrna media	Scoophead Shark		DD
403	Sphyrnidae	Sphyrna mokarran	Great Hammerhead	II	EN
404	Sphyrnidae	Sphyrna tiburo	Bonnethead Shark		LC
405	Sphyrnidae	Sphyrna tudes	Smalleye Hammerhead Shark		VU
406	Sphyrnidae	Sphyrna zygaena	Smooth Hammerhead		VU
407	Squalidae	Cirrhigaleus asper	Roughskin Spiny Dogfish		DD
408	Squalidae	Cirrhigaleus australis	Southern Mandarin Shark		DD
409	Squalidae	Cirrhigaleus barbifer	Mandarin Shark		DD
410	Squalidae	Squalus acanthias	Spiny Dogfish	II	VU
411	Squalidae	Squalus acutirostris	Chinese Longnose Dogfish		DD
412	Squalidae	Squalus albifrons	Eastern Highfin Spurdog		DD
413	Squalidae	Squalus altipinnis	Western Highfin Spurdog		DD
414	Squalidae	Squalus blainville	Longnose Spurdog		DD
415	Squalidae	Squalus brevirostris	Japanese Shortnose Spurdog		DD
416	Squalidae	Squalus bucephalus	Bighead Spurdog		DD
417	Squalidae	Squalus chloroculus	Greeneye Spurdog		NT
418	Squalidae	Squalus crassispinus	Fatspine Spurdog		DD
419	Squalidae	Squalus cubensis	Cuban Dogfish		DD
420	Squalidae	Squalus edmundsi	Edmunds,Äô Spurdog		NT

	Family	Scientific Name	Common Name	CMS Annex (as of Aug 7 2018)	IUCN Red List Status
421	Squalidae	Squalus grahami	Eastern Longnose Spurdog		NT
422	Squalidae	Squalus griffini	Northern Spiny Dogfish		LC
423	Squalidae	Squalus hemipinnis	Indonesian Shortsnout Spurdog		NT
424	Squalidae	Squalus japonicus	Japanese Spurdog		DD
425	Squalidae	Squalus lalannei	Seychelles Spurdog		DD
426	Squalidae	Squalus megalops	Shortnose Spurdog		DD
427	Squalidae	Squalus melanurus	Blacktailed Spurdog		LC
428	Squalidae	Squalus mitsukurii	Shortspine Spurdog		DD
429	Squalidae	Squalus montalbani	Philippine Spurdog		VU
430	Squalidae	Squalus nasutus	Western Longnose Spurdog		DD
431	Squalidae	Squalus notocaudatus	Bartail Spurdog		DD
432	Squalidae	Squalus rancureli	Cyrano Spurdog		NT
433	Squalidae	Squalus raoulensis	Kermadec Spiny Dogfish		LC
434	Squatinidae	Squatina squatina	Angelshark	I/II	CR
435	Torpedinidae	Torpedo nobiliana	Great Torpedo Ray		NA
436	Triakidae	Galeorhinus galeus	Tope		VU
437	Triakidae	Mustelus asterias	Starry Smoothhound		LC
438	Triakidae	Mustelus mustelus	Common Smoothhound		VU
439	Xiphiidae	Xiphias gladius	Swordfish		LC

Appendix 3: Weblinks for online resources

Website listings for processes, databases and repositories mentioned in this paper.

Databases of Area-based Management Tools related to ABNJ

- UN Food and Agriculture Organization's Vulnerable Marine Ecosystems Database
    - www.fao.org/in-action/vulnerable-marine-ecosystems/vme-database
- Ecologically or Biologically Signficant Area (EBSA) Repository
    - https://www.cbd.int/ebsa/

Range maps of species

- Global Registry of Migratory Species
    - http://www.groms.de/
- IUCN
    - http://www.iucnredlist.org/technical-documents/spatial-data
- BirdLife International
    - http://datazone.birdlife.org/home
- AquaMaps (modeled range and distribution maps)
    - http://aquamaps.org
- State of the World's Sea Turtles
    - http://seamap.env.duke.edu/swot

Marine animal tracking data repositories

- United States' Animal Telemetry Network
    - https://atn.ioos.us
- Australia's Integrated Marine Observing System Animal Tracking Database
    - https://animaltracking.aodn.org.au
- BirdLife International's Seabird Tracking Database
    - http://seabirdtracking.org
- OBIS-SEAMAP
    - http://seamap.env.duke.edu
- Ocean Tracking Network
    - http://oceantrackingnetwork.org/
- Movebank
    - http://movebank.org
- Satellite Tracking and Analysis Tool
    - http://www.seaturtle.org

Migratory Connectivity in the Ocean (MiCO)

http://mico.eco/

Appendix 2

Caption Species List: MiCO aims to coalesce existing knowledge on connectivity and migratory corridors of marine migratory species that utilize areas beyond national jurisdiction. Numerous definitions of "migration" and "migratory species" exist, confusing efforts to develop a specific list of species to include within MiCO. Lascelles et al. (2014) assessed the current status and management needs of over 800 migratory marine species across the same four taxonomic groups as MiCO. They define migratory species as animals that move between "at least two jurisdictions during the course of their annual cycles," with specific species selected by expert groups. The list of species to be evaluated by MiCO was assembled from several sources: including Lascelles et al. (2014) (n = 829), the CMS Migratory Shark Species (n = 94), fish species managed by Regional Fisheries Management Organization (RFMO; n = 40), seabirds of the Migratory Bird Treaty Act (n = 171), and BirdLife International (n = 280). The initial 892 species to be evaluated by the MiCO system includes 440 fish, 346 seabird, 99 marine mammal, and 7 sea turtle species.
